# Supplementary material for: Validating the classics: Accurate reference gene panel for reliable RT-qPCR in Porifera
Source: PLoS One. 2026 May 20;21(5):e0349507. doi: 10.1371/journal.pone.0349507 (PMC13189343; doi:10.1371/journal.pone.0349507)
Supplement: S1 File — (DOCX) [file pone.0349507.s001.docx]

# **Supplementary material**

## **Construction of the reference databases**

The *de novo* transcriptome of *E. fluviatilis* was previously published [1]. The *de novo* transcriptome of *L. corallorrhiza* was assembled using Trinity v2.15.2 with standard settings for paired-end reads and read normalization [2]. The resulting transcriptome was processed using CD-HIT-EST [3], with a threshold of 0.95. The final assembly had 405255 transcripts with median and average contig lengths of 308 and 552, respectively; N50 metrics was 637. BUSCO score using eukaryota_odb10 BUSCO set was 98.4% [4].

Genome-based transcriptomes of *H. dujardini*i and *L. hypogea* were retrieved using publicly available genome assemblies (GCA_049997455.1 and GCA_963969325.1, respectively supplemented with our own RNA-seq data for gene annotation. Gene annotation was performed as reported previously [5] using a combination of repeat masking (EarlGrey; [6]), ab initio predictors (BRAKER; [7-9], Trinity *de novo* and genome-guided transcriptome assemblies, StringTie [10] genome assemblies, and miniprot [11] alignment. The resulting genome-derived transcriptomes had BUSCO scores (eukaryota_odb10 BUSCO set) of 92.8 and 90.6%, respectively.

The reads used for the assemblies are available upon request. RNA was extracted using ExtractRNA (BC032, Evrogen, Moscow, Russia), followed by purification using a CleanRNA Standard kit (BC033, Evrogen, Moscow, Russia). Purified RNA samples in RNAse-free water were frozen and stored at -80°C until library preparation. The polyadenylated fraction of the total RNA was purified using the NEBNext Poly(A) mRNA Magnetic Isolation Module (New England Biolabs, Ipswich, MA, USA). Libraries were constructed using the NEBNext Ultra II RNA Library Prep Kit and sequenced using the Illumina NovaSeq6000 (Illumina, San Diego, CA, USA) in paired-end mode with 150 bp read length. The average read number per sample was 40 million. Reads were trimmed using fastp [12] and quality-checked using FastQC (Andrews S. (2010). FastQC: a quality control tool for high throughput sequence data. Available online at: <http://www.bioinformatics.babraham.ac.uk/projects/fastqc>).

The reads were obtained from three independent experimental sets. For *H. dujardinii,* 18 samples from a whole-body regeneration experiment were used [13]. For *L. corallorrhiza*, we used 21 samples from unpublished body-wall regeneration experiments. For *L. hypoge*a, we used an unpublished dataset consisting of 3 samples of intact tissues and 3 cultures of asexually produced buds. The details on the stages and number of replicates are reported in the Table S1.

**Table S1. Sampling summary used in the RNA-seq experiments.**

| **Species** | **Stage/physiological condition** | **No of replicates** |
| --- | --- | --- |
| *Halisarca*  *dujardinii* | Intact tissues | 3 |
|  | Primary multicellular aggregates | 3 |
|  | Early-staged primmorphs | 3 |
|  | True primmorphs | 3 |
|  | Primmorphs with developing aquiferous system, early stage | 3 |
|  | Fully reconstructed sponges | 3 |
| *Leucosolenia*  *corallorrhiza* | Intact tissues | 3 |
|  | 30 min post-operation (po) | 3 |
|  | 1 h po | 3 |
|  | 3 h po | 3 |
|  | 6 h po | 3 |
|  | 12 h po | 3 |
|  | 24h po | 3 |
|  | 48h po | 3 |
| *Lycopodina hypogea* | Intact tissues | 3 |
|  | Asexual buds | 3 |

**Table S2. Total list of transcripts used in the study.**

| **Target** | **Species** | **Sequence name** | **Top pblast hit (UniProtKB & SWISSProt)** | **Blast query (UniProt name & ID)** |
| --- | --- | --- | --- | --- |
| *GAPDH* | *Hdu* | Hdu.evm.model.contig25.17 | A0A238BQ86_9BILA | G3P_MOUSE P16858 |
|  | *Efl* | Efl.m.14207_g.14207 | A0AA35RHM9_GEOBA |  |
|  | *Lhy* | Lhy.evm.model.OZ017791.1.63 | A0AA35RG30_GEOBA |  |
|  | *Lco* | TRINITY_DN8809 | A0A6I9YFP0_9SAUR |  |
| *TBP* | *Hdu* | Hdu.evm.model.contig90.47 | A0A1X7VES3_AMPQE | TBP_MOUSE P29037 |
|  | *Efl* | Efl.m.27899_g.27899 | A0A1X7VES3_AMPQE |  |
|  | *Lhy* | Lhy.evm.model.OZ017782.1.157 | A0A1X7VES3_AMPQE |  |
|  | *Lco* | TRINITY_DN57007_41916 | A0A9J6E132_RHIMP |  |
| *RPL13A* | *Hdu* | Hdu.evm.model.contig30.236 | A0AA35RN17_GEOBA | RL13A_MOUSE P19253 |
|  | *Efl* | Efl.m.11949_g.11949 | A0AA35RN17_GEOBA |  |
|  | *Lhy* | Lhy.evm.model.OZ017787.1.746 | A0AA35RN17_GEOBA |  |
|  | *Lco* | TRINITY_DN12876_134664 | A0AA35RN17_GEOBA |  |
| *RPS3A* | *Hdu* | Hdu.HADA01000668.1 | A0A6P8H362_ACTTE | RS3A_MOUSE P97351 |
|  | *Efl* | Efl.m.1283_g.1283 | RS3A_SUBDO |  |
|  | *Lhy* | Lhy.evm.model.OZ017787.1.307 | RS3A_SUBDO |  |
|  | *Lco* | TRINITY_DN9655_ 124502 | A0A340YEV6_LIPVE |  |
| *HPRT1* | *Hdu* | Hdu.evm.model.contig22.67 | A7STM7_NEMVE | HPRT_MOUSE P00493 |
|  | *Efl* | Efl.m.9314_g.9314 | A0A1X7VL26_AMPQE |  |
|  | *Lhy* | Lhy.evm.model.OZ017788.1.39 | A0A3M6TC76_POCDA |  |
|  | *Lco* | TRINITY_DN1202_3188 | A0A3M6TC76_POCDA |  |
| *RHOA* | *Hdu* | Hdu.evm.model.contig87.143 | A0A672JJP5_SALFA | RHOA_MOUSE Q9QUI0 |
|  | *Efl* | Efl.m.1105_g.1105 | A0A1X7V1M3_AMPQE |  |
|  | *Lhy* | Lhy.evm.model.OZ017790.1.75 | A0AA35R3X6_GEOBA |  |
|  | *Lco* | TRINITY_DN41272 | A0AA88L6H2_ARTSF |  |
| *LMN1* | *Hdu* | Hdu.evm.model.contig32.21 | A0AAN0INZ4_AMPQE | LMNA_MOUSE P48678 |
|  | *Efl* | Efl.m.31108_g.31108 | A0AAN0INZ4_AMPQE |  |
|  | *Lhy* | Lhy.evm.model.OZ017779.1.866 | A0AAN0INZ4_AMPQE |  |
|  | *Lco* | TRINITY_DN9368_99323 | A0AAD7ZQB5_DIPPU |  |
| *ACT1* | *Hdu* | Hdu.evm.model.contig28.48 | ACTC_PISOC | ACTB_MOUSE P60710 |
|  | *Efl* | Efl.m.16996_g.16996 | A2SY09_CIOIN |  |
|  | *Lhy* | Lhy.evm.model.OZ017783.1.600 | A0A913Z1C1_PATMI |  |
|  | *Lco* | TRINITY_DN24_95078 | A7SCN8_NEMVE |  |

Nucleotide sequences are listed in the .fasta file.

**Table S3.** **Description of all primers used to amplify candidate reference genes in the studied species.**

| Gene | Primer sequence (5’-3’) | | PCR  efficiency | Slope | Corr. coefficient (R^2^) | T_a,_ °C | Amplicon size, bp |
| --- | --- | --- | --- | --- | --- | --- | --- |
| *Lco-ACT1* | | F: GTACCACCATGTACCCTGGC | 100,42% | -3,312 | 0,9993 | 57.0 | 192 |
|  |  | R: CGGACTCGTCATACTCCTGC |  |  |  |  |  |
| *Lco-RPL13A* | | F: CATTTGCTTGGTCGTCTGGC | 106.07% | -3,185 | 0,9979 | 59.9 | 85 |
|  |  | R: TGATCTCCTCACACCGGACA |  |  |  |  |  |
| *Lco-GAPDH* | | F: AGGCGTACGGCATTACTGAG | 99,99% | -3,322 | 0,9995 | 57.0 | 238 |
|  |  | R: AGGTCCACAACGGACACATC |  |  |  |  |  |
| *Lco-HPRT1* | | F: AGAACGTCGAAGAGCAGTGG | 100,16% | -3,318 | 0,9967 | 56.7 | 146 |
|  |  | R: GTCGTCTTGCCGAACTCACT |  |  |  |  |  |
| *Lco-RPS3A* | | F: TCCGTCGCAAGATGGTTGAA | 102,61% | -3,261 | 0,9993 | 59.6 | 122 |
|  |  | R: GATGGACTGGCAAGCCTTCT |  |  |  |  |  |
| *Lco-TBP* | | F: GTCAAGTTCTGGCACATCGC | 99,75% | -3,328 | 0,9812 | 58.6 | 151 |
|  |  | R: AGGAGTTGCTGGCATCGAAA |  |  |  |  |  |
| *Lco-LMN1* | | F: TGCCGACGGACTGTTTATCC | 100,97% | -3,300 | 0,9973 | 58.5 | 75 |
|  |  | R: CAGACCACCTTCCAGTGACC |  |  |  |  |  |
| *Lco-RHOA* | | F: CCAGTCAAGCCAGAGGAAGG | 127,05% | -2,808 | 0,9959 | 57.1 | 114 |
|  |  | R: AGTTGCGACTTCGAACACCT |  |  |  |  |  |
| *Hdu-ACT1* | | F: GAGGCTCTCTTCCAGCCATC | 97.24% | -3.39 | 0,9997 | 58.4 | 170 |
|  |  | R: TTCTGCATACGGTCGGCAAT |  |  |  |  |  |
| *Hdu-RPL13A* | | F: TGTCGTTCGGGGGATGATTC | 104.66% | -3.215 | 0.9994 | 60.3 | 85 |
|  |  | R: AGGTGGCATGCCTTCAAAGA |  |  |  |  |  |
| *Hdu-GAPDH* | | F: GACCTCACCTGTCGCTTCAA | 101.35% | -3.29 | 0.9995 | 56.2 | 102 |
|  |  | R: AGTGTAGCCCATCACTCCCT |  |  |  |  |  |
| *Hdu-HPRT1* | | F: GTTCTTCACCGACCTGCTCA | 99.45% | -3.335 | 0.9985 | 56.3 | 202 |
|  |  | R: GCCCTCGTAGCTCTTCAGAC |  |  |  |  |  |
| *Hdu-RPS3A* | | F: AGTCACCAAAACCACTGGCA | 100.71% | -3.305 | 0.9998 | 57.9 | 140 |
|  |  | R: CCCTACCCTGAACCTCCTCA |  |  |  |  |  |
| *Hdu-TBP* | | F: GGTTGGTAGCACCGATGTGA | 97.83% | -3.375 | 0.9849 | 58.0 | 85 |
|  |  | R: CTCAGGCTCGTAACTGGCAA |  |  |  |  |  |
| *Hdu-LMN1* | | F: ACCCTTCGTGATGCAGACAG | 97.04% | -3.395 | 0.9977 | 58.2 | 114 |
|  |  | R: GCGGTCCTTCAGTTCTTGGA |  |  |  |  |  |
| *Hdu-RHOA* | | F: CCTCCGAAACGACGAGAACA | 99.87% | -3.325 | 0.9971 | 59.7 | 155 |
|  |  | R: CAAAGACTTCACGAACGCCC |  |  |  |  |  |
| *Efl-ACT1* | | F: CCGACAGGATGCAGAAGGAG | 101.78% | -3.28 | 0.9990 | 58.0 | 87 |
|  |  | R: CACTGTACTTGCGCTCAGGA |  |  |  |  |  |
| *Efl-RPL13A* | | F: CTACCGCTTTGCGTGTTCTG | 103.09% | -3.25 | 0.9985 | 58.8 | 212 |
|  |  | R: TTCTTGGAGCTGAGGCTTGG |  |  |  |  |  |
| *Efl-GAPDH* | | F: GACTTGACTGTGCGTCTGGA | 99.04% | -3.345 | 0.9995 | 58.0 | 88 |
|  |  | R: CCTTGAGCTGGTCCGATTGT |  |  |  |  |  |
| *Efl-HPRT1* | | F: ATGGCTTCAGACAATGGGCA | 98.23% | -3.365 | 0.9983 | 58.6 | 220 |
|  |  | R: CACACAGGGCTACAAGAGGG |  |  |  |  |  |
| *Efl-RPS3A* | | F: CAGCAATGTTTGCCGTCAGG | 99.87% | -3.325 | 0.9995 | 60.0 | 110 |
|  |  | R: CGCGAGTGACACTTCGAAAA |  |  |  |  |  |
| *Efl-TBP* | | F: AGCACTCCCAGTTCTCAAGC | 100.71% | -3.305 | 0.9990 | 55.8 | 121 |
|  |  | R: TACGTACCGGTCAGCACAAC |  |  |  |  |  |
| *Efl-LMN1* | | F: AGCTGGAAGATCAGGTTGCC | 99.45% | -3.335 | 0.9975 | 57.3 | 230 |
|  |  | R: CCTTAACACACGACGCTCCT |  |  |  |  |  |
| *Efl-RHOA* | | F: GATGGTGCCTGTGGCAAAAC | 102.43% | -3.265 | 0.9993 | 58.6 | 80 |
|  |  | R: AACACGGTGGGAACGTAGAC |  |  |  |  |  |
| *Lhy-ACT1* | | F: GGTCTGGTATGTGCAAGGCT | 104.66% | -3.215 | 0.9995 | 58.4 | 73 |
|  |  | R: CGACCCACGATTGAAGGGAA |  |  |  |  |  |
| *Lhy-RPL13A* | | F: TGGTCCCACACAAGACCAAG | 99.04% | -3.345 | 0.9994 | 58.4 | 132 |
|  |  | R: GCTTCAACTTCAGCACACGG |  |  |  |  |  |
| *Lhy-GAPDH* | | F: TCTTCACGACCACCGACAAG | 100.71% | -3.305 | 0.9991 | 58.4 | 193 |
|  |  | R: ATGACCTTTGCCAGAGGAGC |  |  |  |  |  |
| *Lhy-HPRT1* | | F: TACTACTCCACAGCCAGCCA | 96.84% | -3.4 | 0.9928 | 56.2 | 146 |
|  |  | R: ACCACCAGCACTGTCTTTCC |  |  |  |  |  |
| *Lhy-RPS3A* | | F: ACGAAAGGGAAGAAAGGGGC | 99.04% | -3.345 | 0.9983 | 58.5 | 127 |
|  |  | R: GGGTTCTGGTCACAAGGGTT |  |  |  |  |  |
| *Lhy-TBP* | | F: ATCATTCGCAAGGTCTGCCA | 112.75% | -3.05 | 0.9978 | 58.8 | 166 |
|  |  | R: GAAGCTGAAGCTGAGGGAGG |  |  |  |  |  |
| *Lhy-LMN1* | | F: CACCTACGATGCCAAGCTGA | 104.21% | -3.225 | 0.9976 | 59.7 | 174 |
|  |  | R: TTCACAATCTGGTCGGTCGG |  |  |  |  |  |
| *Lhy-RHOA* | | F: CCGAGCAATGGCAGAGAAGA | 99.25% | -3.34 | 0.9992 | 59.6 | 113 |
|  |  | R: TTGTTTGGAGAGCAGCACGA |  |  |  |  |  |

Primers labelled “F” are forward primers, and primers labelled “R” are reverse primers. Amplification efficiency was determined from each reference gene primer set following RT-qPCR with five 1:10 serial dilutions. One result from three independent runs on different cDNA samples was used to evaluate PCR efficiency.

**Table S4.** Pairwise variation (V values) between combinations of reference genes across experimental groups in *Leucosolenia corallorrhiza*. Calculated using R package ‘ctrlGene’.

| **Pairwise variation**  **(V value)** | **IT**  **(intact tissue)** | **CR**  **(growing regenerative membrane** | **RM**  **(fully sealed regenerative membrane** | **All**  **(total dataset)** |
| --- | --- | --- | --- | --- |
| **V2/V3** | 0.004129084 | 0.004975961 | 0.006926533 | 0.007345737 |
| **V3/V4** | 0.005168201 | 0.005799879 | 0.00709146 | 0.00685507 |
| **V4/V5** | 0.005266468 | 0.004413114 | 0.00537973 | 0.005166656 |
| **V5/V6** | 0.005305304 | 0.003801351 | 0.006511421 | 0.00617398 |
| **V6/V7** | 0.004723175 | 0.003407973 | 0.005400316 | 0.004955452 |

**Table S5.** **Ranking of candidate reference genes in order of expression stability calculated by NormFinder.**

| **Ranking order** | **IT**  **(*Lc0-*)** | **CR**  **(*Lc0-*)** | **RM**  **(*Lc0-*)** | **All**  **(*Lc0-*)** | **IT**  **(*Hdu-*)** | **IT (*Efl-*)** | **IT(*Lhy-*)** |
| --- | --- | --- | --- | --- | --- | --- | --- |
| 1 | *TBP* (0.23) | *RPS3A* (0.16) | *ACT1* (0.31) | *ACT1* (0.27) | *RPL13A* (0.05) | *GAPDH* (0.02) | *RPS3A* (0.12) |
| 2 | *RPS3A* (0.29) | *GAPDH* (0.18) | *HPRT1* (0.45) | *GAPDH* (0.40) | *RPS3A* (0.16) | *ACT1* (0.04) | *HPRT1* (0.19) |
| 3 | *HPRT1* (0.33) | *ACT1* (0.28) | *RPS3A* (0.49) | *HPRT1* (0.41) | *HRPT1* (0.21) | *RPS3A* (0.10) | *GAPDH* (0.21) |
| 4 | *RPL13A* (0.34) | *LMN1* (0.40) | *TBP* (0.51) | *TBP* (0.41) | *ACT1* (0.27) | *LMN1* (0.14) | *LMN1* (0.27) |
| 5 | *ACT1* (0.35) | *TBP* (0.41) | *RPL13A* (0.52) | *RPS3A* (0.43) | *LMN1* (0.30) | *RPL13A* (0.21) | *RPL13A* (0.29) |
| 6 | *GAPDH* (0.35) | *HPRT1* (0.64) | *LMN1* (0.55) | *LMN1* (0.45) | *TBP* (0.44) | *TBP* (0.29) | *ACT1* (0.31) |
| 7 | *LMN1* (0.40) | *RPL13A* (0.74) | *GAPDH* (0.59) | *RPL13A* (0.45) | *GAPDH* (0.49) | *HPRT1* (0.37) | *TBP* (0.55) |

The numbers in brackets indicate stability values.

**Table S6. Normalized *RHOA* gene expression in 3 experimental groups of regeneration model (*L. corallorrhiza*).**

| **Sample** | **Normalized *RHOA* expression** | | | |
| --- | --- | --- | --- | --- |
| **Normalized with:** | ***ACT1*** | ***GAPDH*** | ***RPL13A*** | **combined chosen NF** |
| **IT1** | 1.0 | 1.0 | 1.0 | 1.0 |
| **IT4** | 0.3873093 | 0.467175 | 0.406169 | 0.4188721 |
| **IT5** | 0.649281 | 0.5443564 | 0.5681502 | 0.5855892 |
| **IT6** | 0.382939 | 0.419056 | 0.397944 | 0.3997065 |
| **CR1** | 0.5105912 | 0.751003 | 0.6200936 | 0.619523 |
| **CR2** | 0.4609035 | 0.2888827 | 0.3476838 | 0.3590641 |
| **CR3** | 0.3170017 | 0.552717 | 0.4032166 | 0.4133973 |
| **CR4** | 0.4773852 | 0.4687108 | 0.4688599 | 0.4716346 |
| **CR5** | 0.4421919 | 0.520343 | 0.4396243 | 0.4659372 |
| **CR6** | 0.4287013 | 0.3524498 | 0.376125 | 0.3844691 |
| **RM1** | 0.6870945 | 0.2766888 | 0.4305356 | 0.4341826 |
| **RM2** | 0.8243956 | 0.345112 | 0.5358599 | 0.5342145 |
| **RM4** | 0.4278474 | 0.549826 | 0.4678739 | 0.479234 |
| **RM6** | 0.8576468 | 0.6409332 | 0.7144225 | 0.7323052 |

**
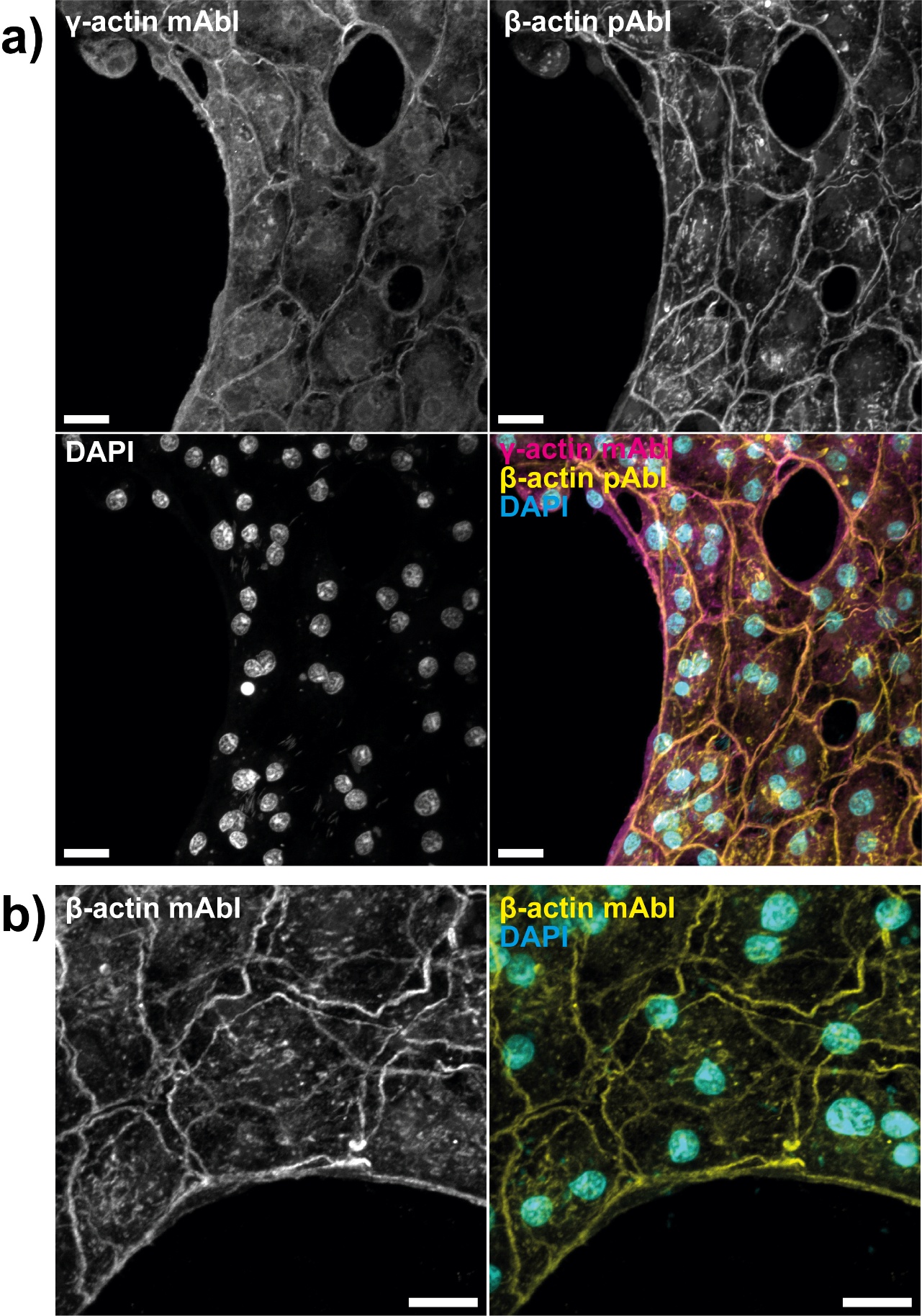
**

**Figure S1. Cytoplasmic actin isoforms immunostaining with different antibodies.** Growing regenerative membrane, *Leucosolenia corallorrhiza*, ~18-22 hours post operation, exo- and endopinacocytes are displayed. MIP of CLSM image. Cyan – DAPI (D9564, Sigma Aldrich), magenta – monoclonal mouse anti-actin-γ antibodies (BioRad MCA 5776GA, clone 2A3), yellow – (a) polyclonal rabbit anti-actin-β antibodies (ab8227, Abcam); (b) monoclonal mouse anti-actin-β antibodies (BioRad MCA5775GA, clone 4C2). Scale bar: 10 μm.

**
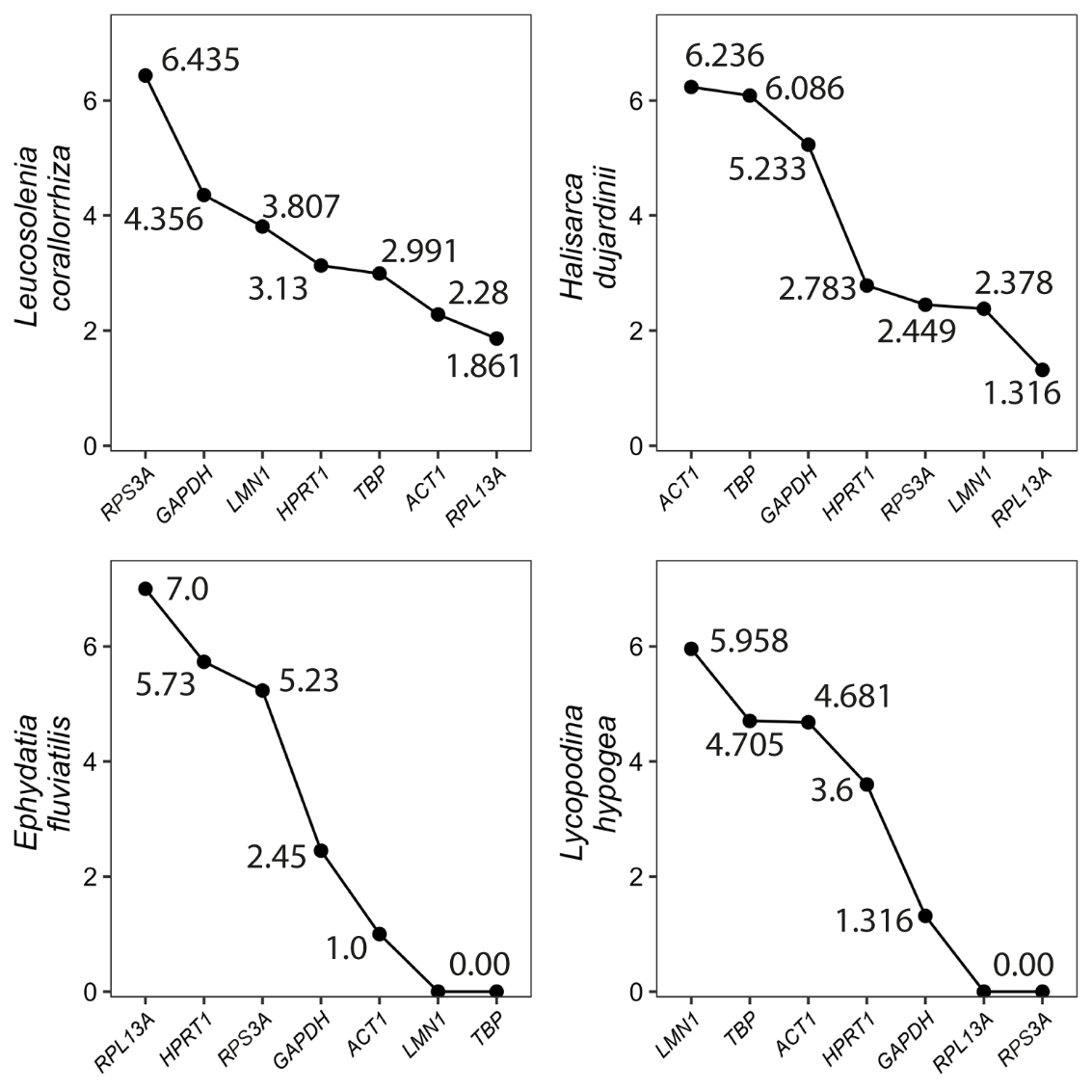
**

**Figure S2. RefFinder stability rankings for candidate reference genes in four sponge species.** Rankings are shown for *Leucosolenia corallorrhiza*, *Halisarca dujardinii*, *Ephydatia fluviatilis*, and *Lycopodina hypogea*. The y-axis represents the RefFinder stability score (lower values indicate higher stability according to the algorithm). Note the biologically implausible ranking of TBP as the most stable gene in *E. fluviatilis*, despite its relatively high variability in other software stability analyses (Fig 5b).

**References**

1. Alié A, Hayashi T, Sugimura I, Manuel M, Sugano W, Mano A, Satoh N, Agata K, Funayama N. The ancestral gene repertoire of animal stem cells. Proc Natl Acad Sci U S A. 2015;112(51):E7093–E7100. doi:10.1073/pnas.1514789112.
2. Grabherr MG, Haas BJ, Yassour M, Levin JZ, Thompson DA, Amit I, Adiconis X, Fan L, Raychowdhury R, Zeng Q, Chen Z, Mauceli E, Hacohen N, Gnirke A, Rhind N, di Palma F, Birren BW, Nusbaum C, Lindblad-Toh K, Friedman N, et al. Full-length transcriptome assembly from RNA-Seq data without a reference genome. Nat Biotechnol. 2011;29(7):644–652. doi:10.1038/nbt.1883.
3. Camacho C, Coulouris G, Avagyan V, Ma N, Papadopoulos J, Bealer K, Madden TL. BLAST+: architecture and applications. BMC Bioinformatics. 2009;10:421. doi:10.1186/1471-2105-10-421.
4. Simão FA, Waterhouse RM, Ioannidis P, Kriventseva EV, Zdobnov EM. BUSCO: assessing genome assembly and annotation completeness with single-copy orthologs. Bioinformatics. 2015;31(19):3210–3212. doi:10.1093/bioinformatics/btv351.
5. Borisenko I, Predeus A, Lavrov A, Ereskovsky A. First draft genome assembly of sponge Halisarca dujardinii reveals key components of basement membrane and broad repertoire of aggregation factors. Sci Rep. 2025;15(1):44778. doi:10.1038/s41598-025-28962-y.
6. Baril T, Galbraith J, Hayward A. Earl Grey: a fully automated user-friendly transposable element annotation and analysis pipeline. Mol Biol Evol. 2024;41(4):msae068. doi:10.1093/molbev/msae068.
7. Gabriel L, Brůna T, Hoff KJ, Ebel M, Lomsadze A, Borodovsky M, Stanke M. BRAKER3: fully automated genome annotation using RNA-seq and protein evidence with GeneMark-ETP, AUGUSTUS and TSEBRA. bioRxiv. 2023:2023.06.10.544449. doi:10.1101/2023.06.10.544449.
8. Brůna T, Lomsadze A, Borodovsky M. GeneMark-ETP significantly improves the accuracy of automatic annotation of large eukaryotic genomes. Genome Res. 2024;34(5):757–768. doi:10.1101/gr.278373.123.
9. Huang, N., & Li, H. (2023). compleasm: a faster and more accurate reimplementation of BUSCO. Bioinformatics (Oxford, England), 39(10), btad595. <https://doi.org/10.1093/bioinformatics/btad595>
10. Kovaka S, Zimin AV, Pertea GM, Razaghi R, Salzberg SL, Pertea M. Transcriptome assembly from long-read RNA-seq alignments with StringTie2. Genome Biol. 2019;20(1):278. doi:10.1186/s13059-019-1910-1.
11. Li H. Protein-to-genome alignment with miniprot. Bioinformatics. 2023;39(1):btad014. doi:10.1093/bioinformatics/btad014.
12. Chen S. Ultrafast one-pass FASTQ data preprocessing, quality control, and deduplication using fastp. iMeta. 2023;2(2):e107. doi:10.1002/imt2.107.
13. Melnikov NP, Skorentseva KV, Ereskovsky AV, Borisenko IE, Bolshakov FV, Lavrov AI. Tissue integrity at the root of Metazoa: transcriptional landscape of whole-body regeneration in sponges. FEBS J. 2026;293(5):1516–1544. doi:10.1111/febs.70337.
