# Supplementary material for: Validating the classics: Accurate reference gene panel for reliable RT-qPCR in Porifera
Source: PLoS One. 2026 May 20;21(5):e0349507. doi: 10.1371/journal.pone.0349507 (PMC13189343; doi:10.1371/journal.pone.0349507)
Supplement: Attachment — Submitted filename: PONE-D-26-03346_converted.pdf [file pone.0349507.s004.pdf]

PLOS One

Validating the classics: accurate reference gene panel for reliable RT-qPCR in Porifera

--Manuscript Draft--

**Manuscript Number:**	PONE-D-26-03346
**Article Type:**	Research Article
**Full Title:**	Validating the classics: accurate reference gene panel for reliable RT-qPCR in Porifera
**Short Title:**	Stable reference genes for RT-qPCR in Porifera
**Corresponding Author:**	Aleena A. Saidova<br>Shenzhen MSU-BIT University<br>Shenzhen, CHINA
**Keywords:**	real-time PCR, reference genes, normalization, Porifera, sponges, non-model organisms
**Abstract:**	Quantitative real-time PCR is a powerful method for quantifying gene expression, but its accuracy depends on reliable normalization strategy. In non-model organisms, like sponges (phylum Porifera), the selection of reliable reference genes is complicated by high variability, seasonal changes, and limited molecular data. We evaluated reference gene candidates in four phylogenetically and ecologically distinct species — Leucosolenia corallorrhiza, Halisarca dujardinii, Ephydatia fluviatilis, and Lycopodina hypogea — representing marine and freshwater, filter-feeding and carnivorous forms. Seven housekeeping genes (ACT1, GAPDH, RPL13A, HPRT1, RPS3A, TBP, LMN1) were identified from transcriptomic and genomic resources, and species-specific primers were designed and validated. Gene expression stability was analyzed using geNorm, NormFinder, BestKeeper, and RefFinder tools under multiple physiological and experimental conditions, including tissue regeneration.<br>RPL13A, ACT1, and GAPDH were the most stable in L. corallorrhiza, and similar rankings appeared in other species, though no single gene was universally stable. Pairwise variation indicated that two reference genes suffice for accurate normalization, while using the geometric mean of three top-ranked genes further improved reproducibility and reduced false positives, as verified with RHOA normalization.<br>Our results provide the first validated panel of reference genes for RT-qPCR in sponges, ensuring robust expression analysis in these early-branching metazoans.
**Order of Authors:**	Kseniia V. Skorentseva
	Nikolai P Melnikov
	Alexander V. Ereskovsky
	Andrey I Lavrov
	Aleena A. Saidova
**Additional Information:**	
**Question**	**Response**
**Financial Disclosure**<br><br>Enter a financial disclosure statement that describes the sources of funding for the work included in this submission. Review the submission guidelines for detailed requirements. View published research articles from *PLOS ONE* for specific examples.	Yes

Powered by Editorial Manager® and ProduXion Manager® from Aries Systems Corporation

This statement is required for submission and **will appear in the published article** if the submission is accepted. Please make sure it is accurate.<br><br>**Funded studies**<br>Enter a statement with the following details:<br>• Initials of the authors who received each award<br>• Grant numbers awarded to each author<br>• The full name of each funder<br>• URL of each funder website<br>• Did the sponsors or funders play any role in the study design, data collection and analysis, decision to publish, or preparation of the manuscript?<br><br>Did you receive funding for this work?	
Please add funding details.<br>as follow-up to "**Financial Disclosure**<br><br>Enter a financial disclosure statement that describes the sources of funding for the work included in this submission. Review the submission guidelines for detailed requirements. View published research articles from *PLOS ONE* for specific examples.<br><br>This statement is required for submission and **will appear in the published article** if the submission is accepted. Please make sure it is accurate.<br><br>**Funded studies**<br>Enter a statement with the following details:<br>• Initials of the authors who received each award<br>• Grant numbers awarded to each author<br>• The full name of each funder<br>• URL of each funder website<br>• Did the sponsors or funders play any role in the study design, data collection and analysis, decision to publish, or preparation of the manuscript?<br><br>Did you receive funding for this work?"	The research was supported by the Russian Science Foundation project no. 23–74–10005 (studies in Leucosolenia corallorrhiza and Halisarca dujardinii) and no. 24-14-00452 (studies in Ephydatia fluviatilis and Lycopodina hypogea).

Please select the country of your main research funder (please select carefully as in some cases this is used in fee calculation).<br>as follow-up to "**Financial Disclosure**<br><br>Enter a financial disclosure statement that describes the sources of funding for the work included in this submission. Review the submission guidelines for detailed requirements. View published research articles from *PLOS ONE* for specific examples.<br><br>This statement is required for submission and **will appear in the published article** if the submission is accepted. Please make sure it is accurate.<br><br>**Funded studies**<br>Enter a statement with the following details:<br>• Initials of the authors who received each award<br>• Grant numbers awarded to each author<br>• The full name of each funder<br>• URL of each funder website<br>• Did the sponsors or funders play any role in the study design, data collection and analysis, decision to publish, or preparation of the manuscript?<br><br>Did you receive funding for this work?"	RUSSIAN FEDERATION - RU
**Competing Interests**<br><br>Use the instructions below to enter a competing interest statement for this submission. On behalf of all authors, disclose any competing interests that could be perceived to bias this work—acknowledging all financial support and any other relevant financial or non-financial competing interests.<br><br>This statement is required for submission and **will appear in the published article** if the submission is accepted. Please make sure it is accurate and that any funding	The authors have declared that no competing interests exist.

sources listed in your Funding Information later in the submission form are also declared in your Financial Disclosure statement.<br><br>View published research articles from *PLOS ONE* for specific examples.<br><br>**NO authors have competing interests**<br><br>Enter: *The authors have declared that no competing interests exist.*<br><br>**Authors with competing interests**<br><br>Enter competing interest details beginning with this statement:<br><br>*I have read the journal's policy and the authors of this manuscript have the following competing interests: [insert competing interests here]*<br><br>* typeset	
**Ethics Statement**<br><br>Enter an ethics statement for this submission. This statement is required if the study involved:<br><br>• Human participants<br>• Human specimens or tissue<br>• Vertebrate animals or cephalopods<br>• Vertebrate embryos or tissues<br>• Field research<br><br>Write "N/A" if the submission does not require an ethics statement.<br><br>General guidance is provided below. Consult the submission guidelines for detailed instructions. **Make sure that all information entered here is included in the Methods section of the manuscript.**	N/A

**Format for specific study types**<br><br>**Human Subject Research (involving human participants and/or tissue)**<br>• Give the name of the institutional review board or ethics committee that approved the study<br>• Include the approval number and/or a statement indicating approval of this research<br>• Indicate the form of consent obtained (written/oral) or the reason that consent was not obtained (e.g. the data were analyzed anonymously)<br><br>**Animal Research (involving vertebrate animals, embryos or tissues)**<br>• Provide the name of the Institutional Animal Care and Use Committee (IACUC) or other relevant ethics board that reviewed the study protocol, and indicate whether they approved this research or granted a formal waiver of ethical approval<br>• Include an approval number if one was obtained<br>• If the study involved *non-human primates*, add *additional details* about animal welfare and steps taken to ameliorate suffering<br>• If anesthesia, euthanasia, or any kind of animal sacrifice is part of the study, include briefly which substances and/or methods were applied<br><br>**Field Research**<br><br>Include the following details if this study involves the collection of plant, animal, or other materials from a natural setting:<br>• Field permit number<br>• Name of the institution or relevant body that granted permission	
**Data Availability**<br><br>Authors are required to make all data underlying the findings described fully available, without restriction, and from the time of publication. PLOS allows rare exceptions to address legal and ethical concerns. See the PLOS Data Policy and FAQ for detailed information.	Yes - all data are fully available without restriction

A Data Availability Statement describing where the data can be found is required at submission. Your answers to this question constitute the Data Availability Statement and **will be published in the article**, if accepted.<br><br>**Important:** Stating 'data available on request from the author' is not sufficient. If your data are only available upon request, select 'No' for the first question and explain your exceptional situation in the text box.<br><br>Do the authors confirm that all data underlying the findings described in their manuscript are fully available without restriction?	
**Describe where the data may be found in full sentences. If you are copying our sample text, replace any instances of XXX with the appropriate details.**<br><br>• If the data are **held or will be held in a public repository**, include URLs, accession numbers or DOIs. If this information will only be available after acceptance, indicate this by ticking the box below. For example: *All XXX files are available from the XXX database (accession number(s) XXX, XXX.*).<br>• If the data are all contained **within the manuscript and/or Supporting Information files**, enter the following: *All relevant data are within the manuscript and its Supporting Information files.*<br>• If neither of these applies but you are able to provide **details of access elsewhere**, with or without limitations, please do so. For example:<br><br>*Data cannot be shared publicly because of [XXX]. Data are available from the XXX Institutional Data Access / Ethics Committee (contact via XXX) for researchers who meet the criteria for access to confidential data.*<br><br>*The data underlying the results presented in the study are available from (include the name of the third party*	A draft dataset supporting the conclusions of this study is available upon request. The R script used to analyze and visualize the data is also available.

and contact information or URL).<br>• This text is appropriate if the data are owned by a third party and authors do not have permission to share the data.<br><br>* typeset	
Additional data availability information:	Tick here if the URLs/accession numbers/DOIs will be available only after acceptance of the manuscript for publication so that we can ensure their inclusion before publication.

Powered by Editorial Manager® and ProduXion Manager® from Aries Systems Corporation

Click here to access/download;Manuscript;Skorentseva 2026.docx

Validating the classics: accurate reference gene panel for reliable RT-qPCR in Porifera

Running title: Stable reference genes for RT-qPCR in Porifera

Authors: Kseniia V. Skorentseva[1], Nikolai P. Melnikov[2,3], Alexander V. Ereskovsky[1,4], Andrey I. Lavrov[3] & Alina A. Saidova[5,6*]

1 – Koltzov Institute of Developmental Biology of Russian Academy of Sciences, 119334, Moscow, Russia

2 – Department of Invertebrate Zoology, Faculty of Biology, Lomonosov Moscow State University, 119991, Moscow, Russia

3 – Pertsov White Sea Biological Station, Faculty of Biology, Lomonosov Moscow State University, 119991 Moscow, Russia

4 – Aix Marseille University, Institut Méditerranéen de Biodiversité et d'Ecologie (IMBE), CNRS, IRD, Avignon University, 13007, Marseille, France

5 – Faculty of Biology, Shenzhen MSU-BIT University, 518000, Shenzhen, China

6 – Department of Cell biology and Histology, Faculty of Biology, Lomonosov Moscow State University, 119991, Moscow, Russia

*Corresponding author: aleena.saidova@gmail.com

Abstract

Quantitative real-time PCR is a powerful method for quantifying gene expression, but its accuracy depends on reliable normalization strategy. In non-model organisms, like sponges (phylum Porifera), the selection of reliable reference genes is complicated by high variability, seasonal changes, and limited molecular data. We evaluated reference gene candidates in four phylogenetically and ecologically distinct species — *Leucosolenia corallorrhiza*, *Halisarca dujardinii*, *Ephydatia fluviatilis*, and *Lycopodina hypogea* — representing marine and freshwater, filter-feeding and carnivorous forms. Seven housekeeping genes (*ACT1, GAPDH, RPL13A, HPRT1, RPS3A, TBP, LMN1*) were identified from transcriptomic and genomic resources, and species-specific primers were designed and validated. Gene expression stability was analyzed using geNorm, NormFinder, BestKeeper, and RefFinder tools under multiple physiological and experimental conditions, including tissue regeneration.

RPL13A, ACT1, and *GAPDH* were the most stable in *L. corallorrhiza*, and similar rankings appeared in other species, though no single gene was universally stable. Pairwise variation indicated that two reference genes suffice for accurate normalization, while using the geometric mean of three top-ranked genes further improved reproducibility and reduced false positives, as verified with *RHOA* normalization. Our results provide the first validated panel of reference genes for RT-qPCR in sponges, ensuring robust expression analysis in these early-branching metazoans.
Key words: real-time PCR, reference genes, normalization, Porifera, sponges, non-model organisms

Introduction

Quantitative real-time PCR (RT-qPCR) is one of the most widely used techniques for measuring gene expression due to its high sensitivity, specificity, and dynamic range. However, obtaining accurate and reproducible results depends critically on the proper normalization of expression data, which compensates for variations in an experimental setup (1-4). This is typically achieved using internal reference genes, which are assumed to maintain stable expression across samples and conditions. Despite the availability of clear guidelines for reference gene validation (5), many studies, especially in invertebrates, rely on housekeeping genes traditionally validated in vertebrate models, without empirical testing in the species of interest. Notably, even in vertebrate systems, it has been shown that reference gene expression may vary significantly across tissues, developmental stages, and experimental conditions (6, 7), thus requiring careful, context-specific validation.

Accurate normalization is especially important in sponges (phylum Porifera), which represent an early-branching metazoan lineage of high evolutionary interest and exhibit considerable biological variability, making the validation of reference genes particularly critical. Sponges possess highly plastic, regeneration-capable tissues, which undergo seasonal, environmental and physiological remodeling, and differ drastically from bilaterians in their cellular architecture and physiology (8-10). The molecular biology of sponges also differs from other animals, as poriferans often show unique composition of many gene families/gene programs (either lacking specific orthologs or undergoing Profera-specific paralogous expansions). Moreover, many sponge species possess a calcareous or siliceous skeleton, which introduces additional biochemical challenges to

RNA extraction and may contribute to residual contaminants, such as calcium ions and polysaccharides, potentially interfering with enzymatic reactions in RT-qPCR (11). Overall, studies on the molecular biology of sponges require proper identification of a reliable set of reference genes specific to this early branching group of metazoans.

Although gene expression studies in sponges are gaining momentum owing to their evolutionary and ecological significance (12-15), systematic validation of reference genes remains virtually absent. Previous RT-qPCR studies in invertebrates have frequently used the ACTB, GAPDH, and ribosomal protein genes, with varying degrees of empirical support (16-20). In Porifera, however, no standardized panels of validated reference genes have been established to date (10, 21), raising concerns regarding the reliability of the expression data generated in this phylum.

To address this gap, we conducted a systematic evaluation of candidate reference genes in the calcareous sponge *Leucosolenia corallorrhiza*, which serves as a model system for regeneration studies. We selected seven widely used candidate genes, *ACT1, RPL13A, GAPDH, HPRT1, RPS3A, TBP,* and *LMN1,* based on their essential roles in cytoskeletal structure, transcription, metabolism, nuclear organization, and ribosomal function. Their expression stability was analyzed across three biologically distinct conditions, reflecting different regeneration stages: intact tissues (IT), body fragments with a growing regenerative membrane (CR), and body fragments with a fully sealed regenerative membrane (RM). To ensure robust assessment, we employed four established computational tools, geNorm, NormFinder, BestKeeper, and RefFinder, and further tested the impact of reference gene selection on the quantification of the regeneration-associated gene *RHOA*. To ensure the broader applicability of the identified reference genes and evaluate their interspecies' stability, we extended the analysis to three additional species representing class Demospongiae and diverse ecological backgrounds: *Halisarca dujardinii* (marine demosponge), *Ephydatia fluviatilis* (freshwater demosponge), and *Lycopodina hypogea* (carnivorous marine demosponge)".

Materials and methods

Sampling

Specimens of *Leucosolenia corallorrhiza* Haeckel, 1870 (Leucosolenida, Calcarea, Porifera) (22) were collected in Kandalaksha Bay, the White Sea, at the outskirts of the

Pertsov White Sea Biological Station of Lomonosov Moscow State University (66°34′ N, 33°8′ E) from June to August 2023. Animals were collected in the upper subtidal zone (0 – 2 m) during low tides and kept in a laboratory aquarium with natural seawater for no longer than five days.

For the regeneration assay in *L. corallorrhiza,* we distinguished three time points: intact tissues (IT); body fragments with a growing regenerative membrane (CR as in «crescent»), 24 hpo; body fragments with a fully sealed regenerative membrane (RM), 48 hpo. IT samples (N = 4) were retrieved by processing whole sponges and clearing debris with minimal damage. As a regeneration model, we used ring-shaped body fragments from oscular tubes. They were cut with Castroviejo scissors, collected, and cleared of debris. To obtain ring-shaped body fragments, oscular tubes were transversely cut into rings 0.5-1.5 mm in width (osculum rims were excluded). All operational procedures were performed manually under a stereomicroscope in 0.22 µm-filtered seawater (FSW). The fragments were sustained in 30 mm plastic Petri dishes with 5 ml of FSW, half of which was changed every 12 h. Body fragments were maintained at physiological temperatures for the species during the summer period (8-12°C). Regular observations under a stereomicroscope were performed to exclude contaminated samples and evaluate the stage of regeneration. Each replicate contained more than 100 ring-shaped fragments obtained from oscular tubes of 1-3 sponges (for CR samples, N = 6, and for RM samples, N = 4).

Specimens of marine sponge *Halisarca dujardinii* Johnston, 1842 (intact tissues, N = 6) were also collected from the upper subtidal zone of Kandalaksha Bay in February 2025. Animals were kept in a laboratory aquarium with natural seawater for no longer than two days.

Gemmules of the freshwater *Ephydatia fluviatilis* Linnaeus (1759) were collected manually from an unnamed small river flowing from the Vaulino Reservoir (v. Vaulino, Sergievo-Posadsky District, Moscow region) in November, 2023. The gemmules were immediately placed in cold water and stored at 2°C until further use. To start germination, gemmules were placed in sterile Mineral-medium, M-medium (1mM $CaCl_2x6H_2O$, 0.5mM $MgSO_4x7H_2O$, 0.5mM $NaHCO_3$, 0.05mM KCl, and 0.25mM Na_2SiO_3) at RT (22°C). Each IT sample (N= 5) consisted of ~40-50 young (7 days after hatching) fully developed sponges.

The culture of the marine carnivorous sponge *Lycopodina hypogea* (Vacelet & Boury-Esnault, 1996) is kept at 2.5 – 3L containers with artificial seawater (14°C) at the Marine Station d'Endoume (Marseille). Under such conditions (weekly feeding with *Artemia salina*

nauplii and water change), the population grows, thrives, and is fully capable of maintaining itself. Adults unfed for at least three weeks were processed as IT samples (*N*=5).

Prior to RNA extraction, all samples were soaked at absorbent paper for a few seconds to dispose of excessive water and lysed in 1 mL of ExtractRNA reagent (BC032, Evrogen, Moscow, Russia), homogenized with polypropylene pestle, incubated for 20 min at RT and stored at -80°C at most for two months.

(*N*=5).

Prior to RNA extraction, all samples were soaked at absorbent paper for a few seconds to dispose of excessive water and lysed in 1 mL of ExtractRNA reagent (BC032, Evrogen, Moscow, Russia), homogenized with polypropylene pestle, incubated for 20 min at RT and stored at -80°C at most for two months.

RNA extraction and reverse transcription

RNA was extracted using the acid guanidinium thiocyanate-phenol-chloroform method. The samples were then incubated with DNAse I (EN052, Thermo Fisher Scientific, Waltham, MA, USA) and the RNAse inhibitor RIboCare (EK005S, Evrogen, Moscow, Russia) for 10 min at 37 °C. RNA was purified and concentrated using a CleanRNAStandard spin-columns kit (BC033, Evrogen, Moscow, Russia) according to the manufacturer's protocol. The RNA concentration was measured spectrophotometrically using a NanoPhotometer (Implen, München, Germany). RNA purity was verified by A260/A280 ratio (median 2.101, range 1.75-2.667) and A260/A230 ratio (median 2.017, range 0.44-3.39). 0.1-1 µg of total RNA were taken for each reverse transcription reaction depending on the species. cDNA was synthesized using Random Primers from RevertAid First Strand cDNA Synthesis Kit (K1621; Thermo Fisher Scientific, Waltham, MA, USA). The required volume of RNA, 1 µL of the manufacturers' Random Hexamer Primers, and nuclease-free water (PB207S, Evrogen, Moscow, Russia) up to 12 µL were mixed. Next, 4 µL of 5x Reaction Buffer, 1 µL of RiboLock RNase Inhibitor (20 units/µL), 2 µL of 10 mM dNTP mix, and 1 µL RevertAid M-MuLV reverse transcriptase (200 units/µL) were added per reaction. The final reaction volume was 20 µL. The tubes were gently mixed and briefly centrifuged. The reaction was incubated for 5 min at 25°C, followed by 60 min at 42°C, and terminated by heating at 72°C for 5 min. Nuclease-free water (80 µL) was added to each reaction mixture, resulting in a final volume of 100 µL. cDNA was stored at -20°C.

Identification of target sequences and primers design

Prospective target sequences were retrieved using tblastn search (23) against the reference databases: a) *de novo* transcriptome of *L. corallorrhiza*; b) genome-derived transcriptome of *H. dujardinii*; b) *de novo* transcriptome of *E. fluviatilis*; c) genome-derived transcriptome of *L. hypogea* (for details on the assemblies see Supplementary Material, Table S1). The query amino acid sequences of *Mus musculus* were obtained using the UniProt database (accessed October-November 2024). Retrieved transcripts were translated using Expasy Translate tool (24) and studied using web-based InterProScan v5.69-101.0 (accessed October-November 2024) to assess the conservation of the encoded protein domain structure. The identification was also validated using reciprocal pblast against the UniProtKB + SwissProt databases.

For lamins, we used a phylogeny-based approach. Amino acid sequences of intermediate filaments from animals other than sponges were retrieved from the UniProt database using manual or blast searches. The resulting set of amino acid sequences was aligned using MUSCLE (25) with default settings; the alignment was trimmed using TrimAl v1.4.1 (26) and used to build maximum-likelihood phylogenetic tree using IQTree web server (27). The ML trees were built using ultrafast bootstrap (n=1000) and SH-aLRT branch support, with automatic assessment of the optimal substitution model. The resulting trees were visualized using FigTree v1.4.4. Nucleic acid and amino acid sequences of target genes/proteins are available at NCBI GenBank/UniProt (see Table S2 for accession IDs).

Primer sequences were designed using the Primer-BLAST Tool (based on Primer3, version 2.5.0) with standard settings for target sequences. All primers were synthesized by Evrogen JCS (Moscow, Russia). The quality of synthesis was manufacturer-controlled by analytical electrophoresis using PAAG and mass spectral analysis. The cDNA samples used in this study were also used in x1, x10, x100, x1000, and x10000 dilutions in triplicates to create a linear regression plot for each gene (in triplicate, the final efficiency percentage is the mean from three independent calculations). Primer details are listed in Table S3.

Real-time quantitative polymerase chain reaction (RT-qPCR)

Real-time PCR was performed in 96-well CFX96 Touch Real-Time PCR Detection System (Bio-Rad, Hercules, CA, USA) or in 96-well DTprime 4M1 PCR Detection System (DNA-Technology, Moscow, Russia) with SYBR Green I dye and reference dye ROX. All reagents were provided by Evrogen (Moscow, Russia) (5X qPCRmix-HS SYBR+High/LowROX, PK155L/PK156L) and the manufacturer's protocols were followed. The reaction volume was 20 μL and contained 4 μL 5x qPCRmix-HS-SYBR+High/LowROX

buffer (includes 3mM $Mg2+$ ions and 0.12mM of each dNTP per each reaction), 25 pmol of each primer, and 2 μL of x5 cDNA dilution. The reaction protocol included denaturation at 95°C followed by 40 amplification cycles – 95°C for 15 s, Ta°C for 30 s, 72°C for 30 s, – and further melting curve analysis (55-95°C, step 0.5°C). All samples were processed in triplicates. The same cDNA sample of cDNA placed repeatedly into each PCR run and served as the inter-run calibrator.

Data analysis and statistics

Relative cDNA quantity (RQ) was calculated as RQ = $CPE^{\Delta CT}$, where

- CPE = $10^{(-1/Eslope)} - 1$; converted PCR efficiency;
- $\Delta CT = Ct_n - Ct_i$; averaged triplicate Ct_n value for each sample, Ct_i – averaged triplicate Ct value for one chosen sample.

The obtained datasets were tested for normality using the Shapiro-Wilk test. Subsequent comparisons were performed using the appropriate statistical tests. The test used is indicated in each case individually. All statistical procedures and data visualization were performed using RStudio version 4.0.3 (RStudio, USA). The significance level (α) was set at < 0.05. The following R packages were used: 'tidyverse', 'ggpubr', 'NormqPCR', 'pastecs'. The R script is available in the Mendeley Data repository (see "Data availability").

Results

To robustly evaluate candidate reference genes for normalization in gene expression studies of sponges, our approach was structured into two major experimental phases. In the first phase, we focused on *Leucosolenia corallorrhiza* to assess the reference gene performance under varying physiological conditions within a single species. Specifically, we established three well-defined experimental groups reflecting the key stages of regeneration: intact tissues (IT), fragments with a growing regenerative membrane (CR; 24 hours post-operation), and fragments with a fully sealed regenerative membrane (RM; 48 hours post-operation). The regenerative model, cell shape transformations, and corresponding cytoskeletal rearrangements were previously described by Skorentseva et al. (28, 29). In this model we assessed the reference gene expression stability across different biological contexts using *RHOA* as the target gene, a marker chosen because of its central role in cytoskeletal reorganization and cell motility, which are key processes in regeneration. Monitoring *RHOA* enabled us to determine whether candidate reference

genes were truly suitable for normalization under various experimental challenges intrinsic to regeneration studies.

In the second phase, we extended our investigation by including three additional sponge species, *Halisarca dujardinii*, *Ephydatia fluviatilis*, and *Lycopodina hypogea,* to test the universality of the candidate reference genes identified in *L. corallorrhiza*. By comparing these reference genes across evolutionarily and ecologically distinct species, we aimed to determine whether consistent candidate genes could serve as reliable normalizers, regardless of the taxonomic background.

Identification of target sequences

The identification of *GADPH*, *TBP*, *RPS3A*, *RPL13A*, *HPRT1*, and *RHOA* did not need to be supplemented by phylogenetic trees because a single copy for each gene/transcript was found in the transcriptomes. The results of reciprocal BLAST searches and the predicted domain structures of proteins encoded by the studied genes/transcripts (Figure 1a-h, Table S2) unambiguously validated the identification.

For lamins, several prospective sequences were identified. All of them clearly fell into the clade containing lamins, rather than cytoplasmic intermediate filaments (Figure 2). Poriferan lamins tended to cluster together with sequences from cnidarians, and based on the topology of the tree, it seems that calcarean and demosponge lamins are highly divergent (Figure 2). We found a single lamin-coding sequence in the *L. hypogea* and *E. fluviatilis* transcriptomes. For *H. dujardinii* and *L. corallorrhiza*, we chose transcripts that encoded proteins with less predicted disorder (namely, Hdu_evm.model.contig32.21 and Lco_TRINITY_DN9368_99323) as targets for RT-qPCR.

We identified several actin-coding sequences in each database. Actin-like proteins were filtered using reciprocal blast. However, the precise phylogenetic relationships between the remaining actins remained unclear, as the attempt to construct ML trees resulted in low node support values. Thus, we aligned found sequences to β- and γ - cytoplasmic isoforms of vertebrates; contigs Lco_TRINITY_DN24_95078 (*L. corallorrhiza*), Hdu_evm.model.28.48 (*H. dujardinii*), Lhy_evm.model.OZ017783.1.600 (*L. hypogea*), and Efl_ m.16996_g.16996 (*E. fluviatilis*) encoded proteins with the highest similarity to vertebrate *ACTB* and were chosen for the subsequent analysis with *ACT1* name (Figure 3). Notably, the predicted *E. fluviatilis* sequence was fragmented and lacked the proper N-end of the amino acid chain. Based on the amino acid residues at the conserved sites, the

respective proteins resembled the vertebrate β-cytoplasmic actin isoform (Figure 3a-c). Supposedly, the identified contigs encode some form of cytoplasmic actins in sponges: immunostaining of *L. corallorrhiza* regenerative membranes with monoclonal mouse anti-actin-β (BioRad MCA5775GA, clone 4C2), monoclonal mouse anti-actin-γ antibodies (BioRad MCA 5776GA, clone 2A3), and also polyclonal rabbit anti-actin-β antibodies (ab8227, Abcam) (Figure S1) resulted in staining cytoplasmic filamentous structures and stress fibres with a certain level of differential distribution of isoforms.

Expression variability and stability assessment of candidate reference genes in Leucosolenia corallorrhiza

We conducted a comprehensive assessment and evaluation of candidate reference genes in *L. corallorrhiza* using four established algorithms: geNorm, NormFinder, BestKeeper, and RefFinder. Based on the analysis of existing data on the assessment of gene expression in invertebrates using real-time PCR, we selected seven candidate reference genes, *ACT1, RPL13A, GAPDH, HPRT1, RPS3A, TBP,* and *LMN1*, which are commonly used in normalization studies because of their involvement in essential cellular processes, such as cytoskeletal organization (*ACT1*), energy metabolism (*GAPDH*), ribosomal structure (*RPL13A, RPS3A)*, transcription regulation (*TBP*), nucleotide metabolism (*HPRT1*), and nuclear integrity (*LMN1*).

To assess the variability in the expression of the selected genes, we first analyzed their cycle threshold (Ct) values (N=14). Expression analysis of the seven candidate reference genes across all samples revealed significant differences in both expression levels and variability (Figure 4a, Table 1). The coefficients of variation (CV) for all genes remained below 0.04, suggesting relatively stable expression in biological replicates. The lowest Ct values, corresponding to the highest expression, were observed for *ACT1*, whereas *TBP* exhibited the highest Ct values, indicating a lower transcript abundance. *RPL13A* and *RPS3A* showed intermediate and relatively stable expression, as reflected by their narrow interquartile ranges and limited number of outlier points. In contrast, *HPRT1* and *TBP* demonstrated pronounced variability, with more outliers (in case of *TBP*), suggesting less consistent expression across the samples. Next, we plotted histograms with fitted curves to show the distribution of the Ct values for each gene (Figure 4b). *ACT1*, *RPL13A,* and *GAPDH* displayed approximately normal (unimodal and symmetric) Ct value distributions, indicating uniform gene expression within the dataset. Meanwhile, *HPRT1* and TBP showed

more skewed or multimodal distributions, reflecting greater instability and potential condition-dependent expression fluctuations.

To evaluate the stability of the selected reference genes, we performed an analysis using the geNorm, NormFinder and BestKeeper applications (Figure 4c). The geNorm algorithm evaluates the stability of the reference gene by calculating the M value, which represents the average pairwise variation of a gene relative to all the other tested genes. Genes with lower M values are considered more stable. Our results showed that *RPL13A, RPS3A* and *GAPDH* had the lowest M values, indicating the highest stability, whereas *TBP* and *LMN1* exhibited the highest variability. In addition to ranking genes, geNorm calculates pairwise variation (V value, the average variation between the normalization factors using n and n+1 reference genes, indicating the effect of adding an extra gene on normalization accuracy) to determine the minimum number of genes required for reliable normalization. The algorithm assumes that the most stably expressed genes should have minimal variation relative to each other, with a recommended threshold of 0.15. The analysis of pairwise variation coefficients demonstrated consistently low V values across all experimental groups (Figure 4d). The V2/3 values for all conditions, including intact tissues, growing regenerative membranes, and fully sealed regenerative membranes, were well below the 0.15 threshold, indicating that the use of only two reference genes was sufficient for reliable normalization. This confirms that the selected reference genes exhibit strong co-expression stability and that further inclusion of additional genes does not significantly improve the normalization accuracy.

The NormFinder algorithm ranks candidate reference genes based on their stability value, which is determined by estimating intragroup (within experimental conditions) and intergroup (between conditions) variations. Unlike geNorm, which relies on pairwise comparisons, NormFinder calculates the systematic variance of each gene independently and considers the effects of experimental grouping. Genes with lower stability values were more reliable for normalization. According to NormFinder, *ACT1* was identified as the most stable gene, followed by *GAPDH* and *TBP*, whereas *RPL13A* and *LMN1* showed higher variability (Figure 4c, Table S4).

The BestKeeper algorithm assesses reference gene stability based on their standard deviation (SD) and coefficient of variance (CV). Genes with lower SD and CV were considered more stable. Unlike geNorm and NormFinder, BestKeeper does not assume equal expression ratios between genes but instead evaluates absolute Cq values directly. In

our analysis, *GAPDH*, *RPL13A,* and *ACT1* had the lowest SD values, suggesting that they were the most stable genes according to BestKeeper, whereas *HPRT1* and *LMN1* had the highest SD values, indicating significant variability (Figure 4c). Given that *RPL13A*, *ACT1,* and *GAPDH* consistently appeared among the top three across three independent algorithms (ranked top-3 at least by two of the three algorithms) (Table 2), and considering their normal distribution and low variability in expression, using these genes provides robust and reliable normalization across different experimental conditions in *Leucosolenia corallorrhiza*.

Cross-species evaluation of reference gene stability

To assess the universality and robustness of the identified candidate reference genes, we extended our analysis to three additional sponge species representing diverse taxonomic and ecological backgrounds: *Halisarca dujardinii* (marine demosponge), *Ephydatia fluviatilis* (freshwater demosponge), and *Lycopodina hypogea* (carnivorous marine demosponge). This comparative approach enabled us to determine whether consistent reference genes could serve as reliable normalizers across different sponge taxa, thereby providing insights into the broader applicability of our findings for quantitative gene expression studies in Porifera.

In *H. dujardinii*, geNorm identified *RPS3A* and *HPRT1* as the most stable genes, *ACT1* and *RPL13A* also demonstrated high stability. NormFinder ranked *RPL13A*, *RPS3A*, and *HPRT1* as the top candidates, whereas BestKeeper analysis confirmed the high stability of *RPL13A, RPS3A*, and *TBP*. Collectively, *RPS3A, RPL13A,* and *HPRT1* were the most reliable reference genes for this species (Figure 5a, Table 2).

Both geNorm and NormFinder indicated that *GAPDH, ACT1,* and *RPS3A* were the most stably expressed genes in *E. fluviatilis*. The BestKeeper analysis was consistent, highlighting *RPS3A, ACT1,* and *HPRT1* as the top performers. Thus, *ACT1, RPS3A,* and *GAPDH* were determined as the most suitable reference genes for this species (Figure 5b, Table 2).

For *L. hypogea*, geNorm suggested *GAPDH* and *RPL13A* as the most stable pair, followed by *ACT1* and *RPS3A*. NormFinder ranked *RPS3A*, *HPRT1,* and *GAPDH* as the most stable genes, while BestKeeper analysis favored *RPL13A*, *ACT1*, and *LMN1*. Based on these results, *GAPDH*, *RPL13A*, *RPS3A*, and *ACT1* were identified as the most stable genes in *L. hypogea* (Figure 5c, Table 2).

To compare our multi-algorithm approach with a commonly used consensus tool, we also analyzed candidate reference genes using RefFinder. The resulting rankings are shown in Figure S2. RefFinder integrates the outputs of four commonly used algorithms (geNorm, NormFinder, BestKeeper, and the ΔCt method) by calculating their geometric mean to generate a consensus stability ranking of candidate reference genes (30). The RefFinder consensus rankings of candidate reference genes showed some species-specific variation and in several cases diverged from the stability patterns obtained with individual algorithms. In *L. corallorrhiza*, RefFinder placed *RPL13A* and *ACT1* at the top, while RPS3A appeared least stable. However, in our multi-algorithm evaluation, both RPS3A and RPL13A were consistently among the most stable genes.

In *H. dujardinii*, RefFinder ranked LMN1 and RPL13A as the most stable genes. This contrasted all other outputs, as none of the algorithms ranked LMN1 among the most stable.

In *E. fluviatilis*, RefFinder identified TBP as the most stable gene2, but surprisingly placed RPL13A and HPRT1 at the very bottom. This was biologically implausible, since TBP displayed relatively high variability across all other algorithms, and none of the independent methods supported such instability. These comparisons demonstrate that RefFinder may over- or under-estimate the stability of certain genes, producing rankings that contradict other algorithmic outputs.

Taken together, comparative analysis across all three species indicated that the most consistently stable reference genes included a ribosomal gene (*RPL13A* or *RPS3A*), *ACT1*, and *GAPDH*. This combination provides a reliable basis for normalization in gene expression studies involving diverse sponge species (Table 2).

Effect of single vs. multiple reference gene normalization on RHOA expression

Using the three selected reference genes individually for normalization resulted in notably different *RHOA* expression values, whereas the combined normalization factor (NF) yielded more consistent measurements. As shown in Figure 6a, single-gene normalization can over- or underestimate *RHOA* levels for the same sample. For example, the *RHOA* expression normalized to *ACT1* was higher than that normalized to *GAPDH* or *RPL13A*. Such disparities indicate how much using a single reference gene can bias the results. In contrast, when using the combined NF, the *RHOA* expression of each sample fell between the extremes of the single-gene values, resulting in a more moderate and uniform

estimate. This pattern was consistent across all experimental conditions in the regeneration assay for *L. corallorrhiza*.

The scatter plot panels reinforce that normalization with multiple reference genes reduces variability. For *L. corallorrhiza* (Figure 6b), normalization to a single gene, even to a commonly used and relatively stable gene such as *ACT1*, results in greater variability and can lead to significant differences in gene expression between treatments. In *L. hypogea*, the expression values for the same treatment can differ by up to 1.5-fold, depending on the reference gene used (Figure 6c). This variability reflects the sensitivity of single-gene normalization to inter-sample variation and occasional outliers. In contrast, normalization with combined NF produces values that exhibit significantly smaller standard deviations. Similar trends were evident in *H. dujardinii* and *E. fluviatilis*: the single-reference normalized values varied considerably, whereas the NF-normalized values clustered tightly, indicating consistent expression.

Discussion

Gene expression analysis has long become an essential tool in contemporary biological research, providing insights into molecular mechanisms across a wide range of organisms. Although significant progress has been made in developing methods for assessing gene expression, most protocols and normalization strategies have been optimized for model organisms or specific experimental setups. Extending these approaches to complex and evolutionarily distinct groups, such as Porifera (sponges), presents unique challenges. In particular, the selection and validation of appropriate normalization methods and reference genes remain problematic, as much of the literature is dedicated to single-object studies or relies on housekeeping genes, whose universality is questionable in such distant taxa (31-34). Therefore, addressing gene expression in Porifera requires a critical re-examination of methodological strategies, ensuring that analyses adequately reflect the evolutionary and morphological diversity of this group, rather than being constrained by the limitations of standard single-gene approaches.

In non-model phylogenetic groups, difficulties with gene expression normalization often begin at the first stage identifying correct candidate reference gene sequences. Errors in this step may result in the selection of incomplete, misannotated, or paralogous sequences that are not true functional orthologs of the intended genes. This, in turn, can

distort RT-qPCR results, as the apparent expression stability of such sequences may reflect artifacts of incorrect identification rather than genuine biological consistency. In several studies on invertebrates and other non-model taxa (35-38), detailed sequence validation was not explicitly discussed, and BLAST search results were used directly without additional confirmation of orthology, domain structures, or phylogenetic placement. Such a simplified approach can lead to the misinterpretation of normalized data, particularly in groups where gene families are highly divergent, contain multiple copies, and have taxon-specific isoforms. Therefore, comprehensive verification, including phylogenetic analysis, domain structure assessment, and conserved motif searches, is essential for accurate selection of reference genes in non-model organisms.

Initial screening of *L. corallorrhiza* identified ribosomal protein genes (*RPL13A and RPS3A*) as well as *GAPDH* and *ACT1* as the most stable candidates. Ribosomal genes are often recommended for normalization because of their fundamental roles in protein synthesis and their presumed consistent expression. Similarly, traditional housekeeping genes such as *ACT1* and *GAPDH* demonstrated stable performance in our dataset. However, previous research in other metazoans has shown that the expression of these genes can be highly context-dependent and may vary with tissue type, developmental stage, or experimental treatment (31, 39-41). Our findings confirm that, while such genes are suitable for specific experimental setups, such as regeneration in *L. corallorrhiza,* they should not be assumed to be universally stable.

Three commonly used algorithms, geNorm, NormFinder and BestKeeper, were employed to assess expression stability. Similar to other studies, some discrepancies in gene rankings were observed across the algorithms. For example, while NormFinder ranked *ACT1* as the most stable gene, geNorm ranked it lower. These inconsistencies underscore the importance of integrated approaches. We also tested RefFinder as a consensus-based ranking tool but ultimately decided not to include its outputs in the final results. During our analysis, we observed that RefFinder sometimes produced biologically implausible rankings. For example, in *Ephydatia fluviatilis*, it ranked TBP as the most stable gene, despite this gene showing relatively high variability across all other stability assessments (Figure S1). Similar issues have been documented previously, for instance, de Spiegelaere et al. (42) reported that RefFinder does not take PCR amplification efficiencies into account, which can bias the rankings. This limitation is particularly critical in non-model

species, where PCR efficiency can vary significantly between targets due to sequence divergence, and uncorrected data may therefore lead to misleading conclusions.

Our analysis further demonstrated that normalization using a combination of three genes, namely RPL13A, ACT1, and GAPDH, provides robust and reproducible normalization in *L. corallorrhiza*. These results are consistent with the MIQE (Minimum Information for Publication of Quantitative Real-Time PCR Experiments) guidelines, which recommend the use of multiple validated reference genes (1, 5).

Extension of the analysis to three additional sponge species revealed considerable interspecies variability in reference gene stability. No single gene was universally stable across all of the four species. For example, *RPL13A*, highly ranked in *L. corallorrhiza*, showed lower stability in *H. dujardinii*, whereas *TBP*, unstable in *L. corallorrhiza*, ranked higher in other species. Such interspecies variations in gene expression stability should be taken in account during experiment planning.

The consequences of the reference gene selection were further illustrated by our analysis of *RHOA* expression. *RHOA* is a key regulator of cytoskeletal remodeling and cell motility(43), making it a biologically relevant target during regeneration. However, when normalized to a single reference gene, even to a relatively stable one such as *ACT1,* we observed significant variability across samples and treatments, which could result in false-positive or false-negative conclusions. In contrast, normalization using the geometric mean of *RPL13A, ACT1,* and *GAPDH* mRNA quantities significantly reduced variability and yielded more consistent and biologically meaningful expression patterns.

Conclusion

We identified robust reference gene combinations for RT-qPCR normalization in diverse sponge species, emphasizing the need for sequence-level verification and multi-algorithm stability assessment. In *Leucosolenia corallorrhiza*, *RPL13A, ACT1,* and *GAPDH* provided the most consistent performance, and similar patterns were observed in other species. Multi-gene normalization markedly reduced variability and prevented misleading interpretations, as demonstrated for *RHOA* expression. These results offer a validated framework for accurate and reproducible gene expression studies in Porifera.

List of abbreviations

ACT1– β-cytoplasmic actin-like gene; CR – crescent regenerative membrane; CV – coefficient of variation; Efl – *Ephydatia fluviatilis*; FSW – filtered seawater; *GAPDH* –

glyceraldehyde 3-posphate dehydrogenase; hpo – hours post-operation; Hdu – *Halisara dujardinii*; *HPRT1* – hypoxanthine phosphoribosyl-transferase 1; IT – intact tissue; Lco – *Leucosolenia corallorrhiza*; Lhy – *Lycopodina hypogea*; LMN1 – lamin gene; NF – normalization factor; *RHOA* – Ras homolog family member A; *RPL13A* – ribosomal protein L13a; *RPS3A* – 40S ribosomal protein S3a; RT-qPCR – Quantitative real-time PCR; SD – standard deviation; *TBP* – TATA box binding protein.

Limitations

Several factors should be considered when interpreting the results of this study. First, although RNA integrity and purity were carefully monitored, the inherent challenges of RNA extraction from sponge tissues — such as the presence of mineral skeleton components and polysaccharides — may have introduced variability in template quality. Second, for some experimental groups, the number of biological replicates was limited by specimen availability, potentially reducing the robustness of statistical comparisons. Third, while the use of multiple validated reference genes improves normalization accuracy, it also increases the experimental workload and resource demands, which may limit the feasibility of this approach in large-scale or time-sensitive studies. Finally, the stability of reference genes can be influenced by the specific experimental condition and tissue type analyzed; therefore, the suitability of the selected genes should be re-evaluated when experimental designs deviate substantially from those tested here.

Acknowledgements

The authors acknowledge the support of the Lomonosov Moscow State University Program of Development (DTprime 4M1 PCR). KS acknowledges Ostrogradski scholarship 2024 (French Embassy in Russia) in frame of which studies of *L. hypogea* were conducted in the University of Montpellier. The authors sincerely thank Julia Kraus ant Anton Bogomolov (Koltzov IDB RAS), Emilie Le Goff, Nelly Godefroy and Stephen Baghdiguian (Université Montpellier, France) for continuous assistance regarding sponge maintenance and helpful advice. The authors are grateful to the Core Facility Centrum of the Koltzov Institute of Developmental Biology RAS supported by the IDB RAS research program №0088-2024-0012 for the possibility to use the equipment.

Conflict of interests

The authors declare that they have no conflict of interest.

Author contributions

KS and AS designed the study. KS performed all the experimental procedures regarding sponge maintenance, RNA extraction, and RT-qPCR analysis. NM performed bioinformatics analysis. KS and AS wrote the manuscript with contributions from all the authors. AS, AE and and AL revised and edited the final manuscript. All authors have reviewed and approved the final manuscript.

Data availability statement

A draft dataset supporting the conclusions of this study is available upon request. The R script used to analyze and visualize the data is also available.

Funding

The research was supported by the Russian Science Foundation project no. 23–74–10005 (studies in *Leucosolenia corallorrhiza* and *Halisarca dujardinii*) and no. 24-14-00452 (studies in *Ephydatia fluviatilis* and *Lycopodina hypogea*).

Figures

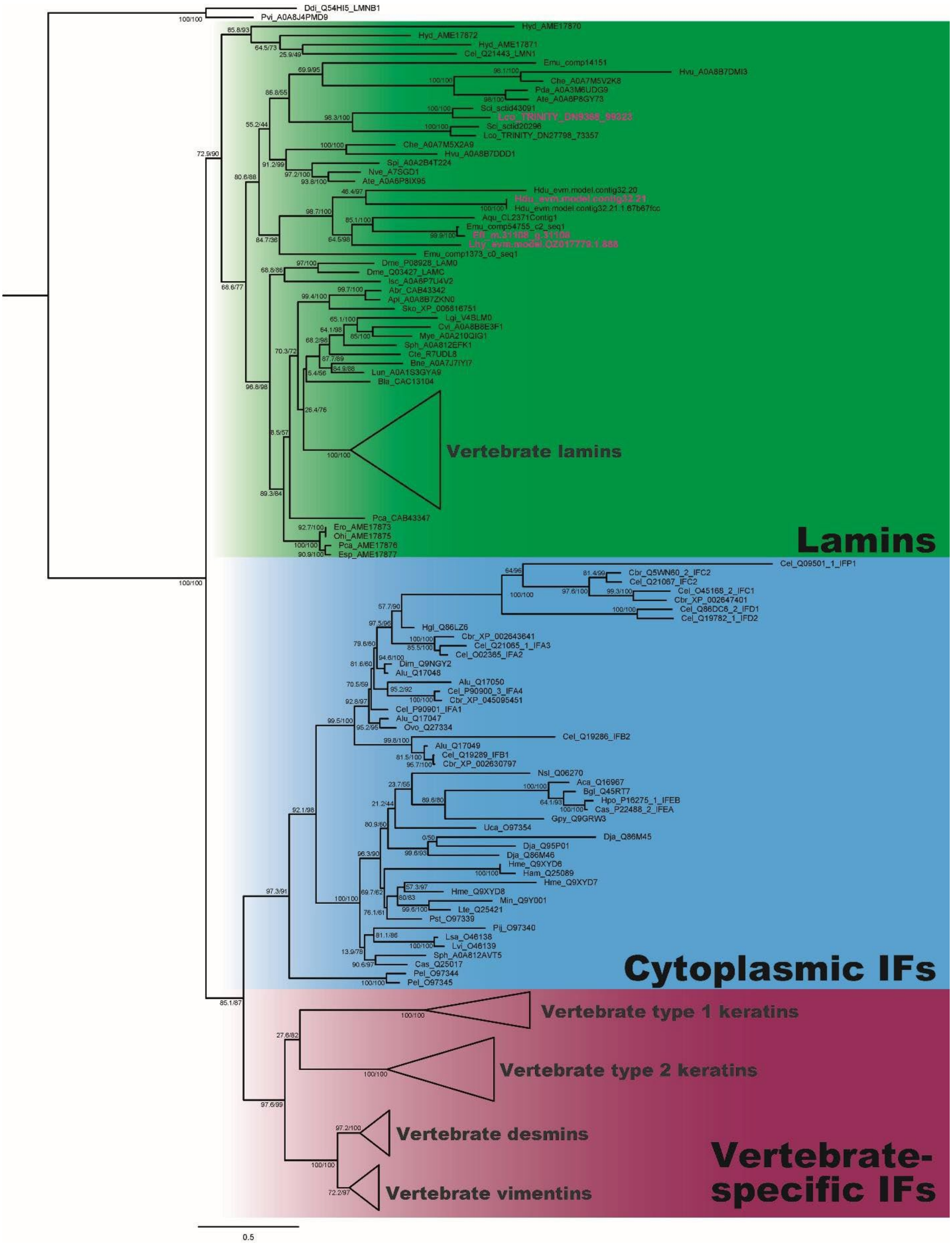

Figure 1. Domain structures of proteins encoded by the identified transcripts. GAPDH (A), RPS3A (B), RPL13A (C), HPRT1 (D), RHOA (E), LMN1 (F), ACT1 (G), TBP (H).

Hdu – *Halisarca dujardinii*, Lhy – *Lycopodina hypogea*, Efl – *Ephydatia fluviatilis*, Lco – *Leucosolenia corallorrhiza*.

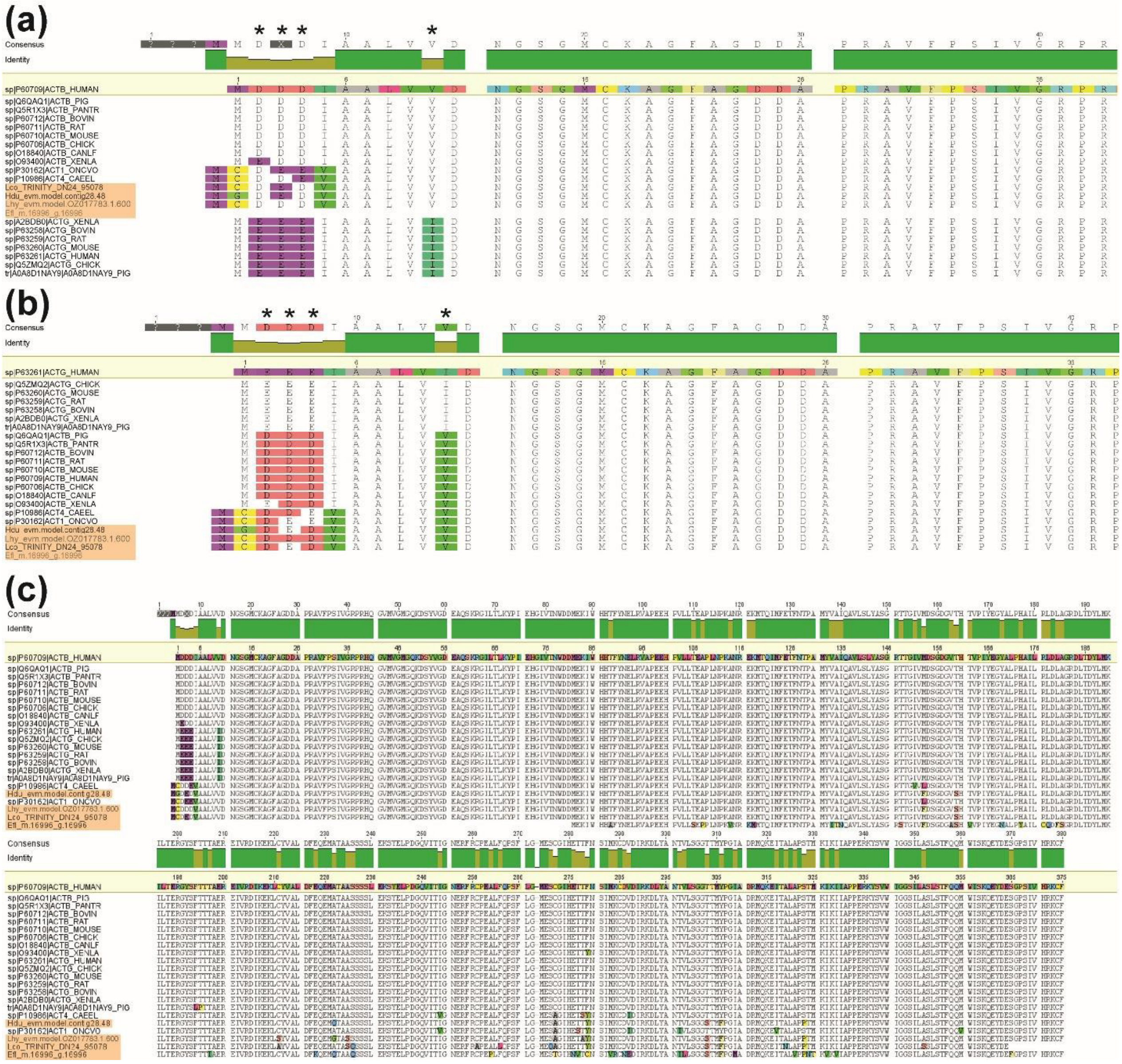

Figure 2. Phylogenetic relationships among intermediate filament (IF) proteins. A maximum likelihood tree was constructed using IQTree with ultrafast bootstrap (n=1000) and SH–aLRT branch support. A full-length alignment trimmed with TrimAl was used to construct the tree. The substitution model LG+G4 was chosen automatically. Node labels represent ultrafast bootstrap/SH–aLRT support values. Abr – *Astropecten brasiliensis*, Aca – *Aplysia californica*, Apl – *Acanthaster plancii*, Aqu – *Amphimedon queenslandica*, Ate – *Actinia tenebrosa*, Alu – *Ascaris lumbricoides*, Aam – *Amphibalanus amphitrite*, Bgl – *Biomphalaria glabrata*, Bne – *Bugula neritina*, Cas – *Cornu aspersum*, Cel – *Caenorhabditis elegans*, Cbr – *Caenorhabditis briggsae*, Che – *Clytia hemisphaerica*, Cin – *Ciona intestinalis*,

Csa – *Ciona savignyi,* Cte – *Capitella teleta,* Cvi – *Crassostrea virginica*, Ddi – *Dictyostelium discoideum*, Dme – *Drosophila melanogaster*, Dpu – *Daphnia pulex*, Dim – *Dirofilaria immitis*, Dja – *Dugesia japonica*, Emu – *Ephydatia muelleri*, Efl – *Ephydatia fluviatilis*, Ero – *Euperipatoides rowelli*, Esp – *Eoperipatus* sp. LH–2012, Gpy – *Glottidia pyramidata*, Ham – *Haemopis marmorata*, Hex – *Hypsibius dujardini*, Hdu – *Halisarca dujardinii*, Hgl – *Heterodera glycines*, Him – *Hirudo medicinalis*, Hpo – *Helix pomatia*, Hvu – *Hydra vulgaris*, Isc – *Ixodes scrapularis*, Lco – *Leucosolenia corallorrhiza*, Lgi – *Lottia gigantea*, Lun – *Lingula unguis*, Lte – *Lumbricus terrestris*, Lhy – *Lycopodina hypogea*, Lsa – *Lineus sanguineus*, Lvi – *Lineus viridis*, Min – *Myxicola infundibulum*, Mye – *Mizuhopecten yessoensis*, Nve – *Nematostella vectensis*, Nsl – *Nototodarus sloanii*, Ohi – *Ooperipatus hispidus*, Osp – *Oscarella* sp., Ovo – *Onchocerca volvulus*, Pel – *Parasagitta elegans*, Phi – *Principapillatus hitoyensis*, Pca – *Priapulus caudatus*, Pij – *Phoronis ijimai*, Pst – *Phascolion strombus*, Pvi – *Polysphondylium violaceum*, Sci – *Sycon ciliatum*, Spi – *Stylopora pistillata*, Sph – *Sepia pharaonis*, Sko – *Saccoglossus kowalewski*, Uca – *Urechis caupo*.

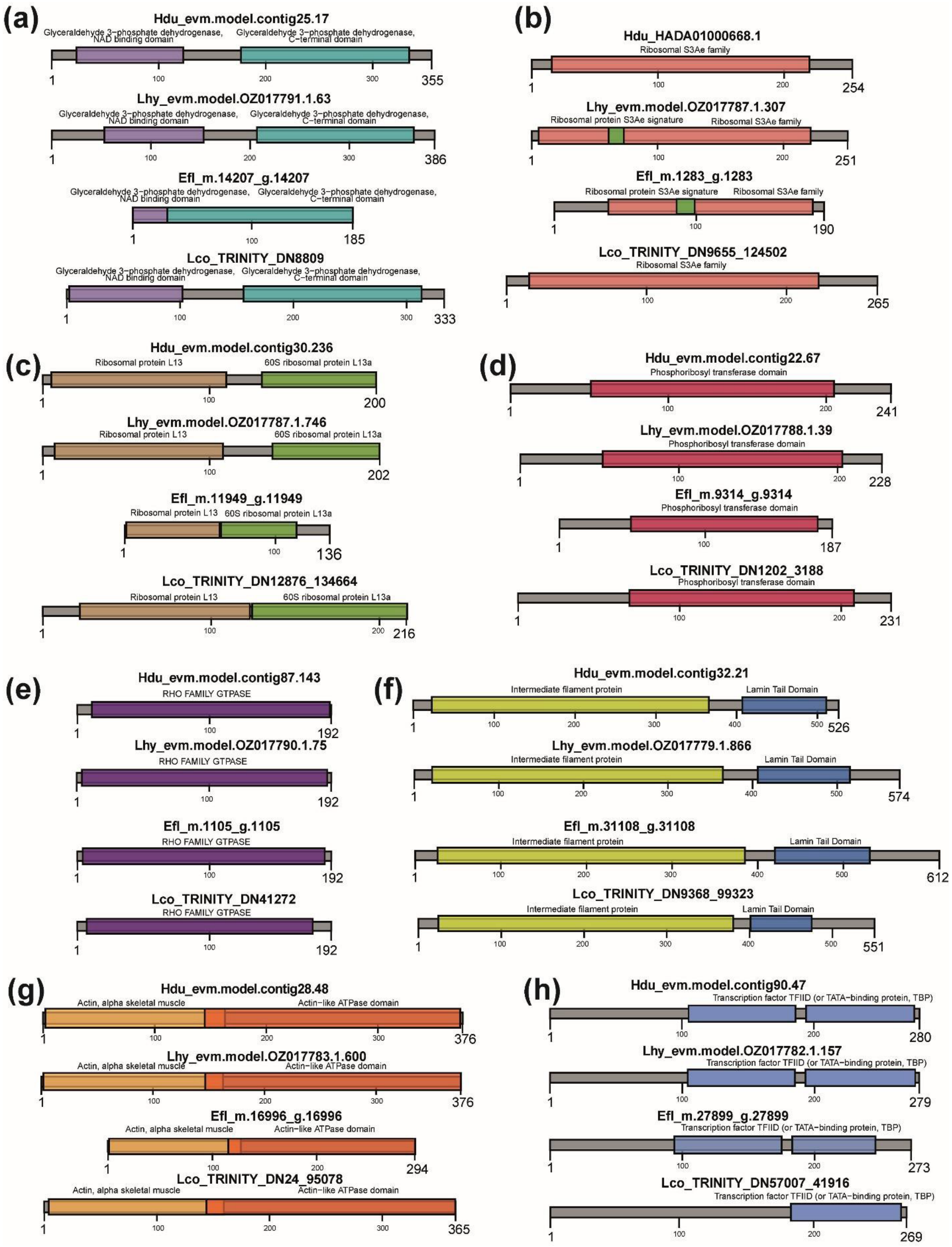

Figure 3. Alignment of amino acid sequences encoded by the identified transcripts. A, B – N-end of the alignment with (, respectively) human β-cytoplasmic actin and γ-cytoplasmic actin set as reference sequences. Colored residues outside the reference sequence represent residues that deviate from the reference sequence. Asterisks indicate

amino acid positions essential for distinguishing between β- and γ-actin in vertebrates. C – the whole alignment with human β-cytoplasmic actin was set as the reference.

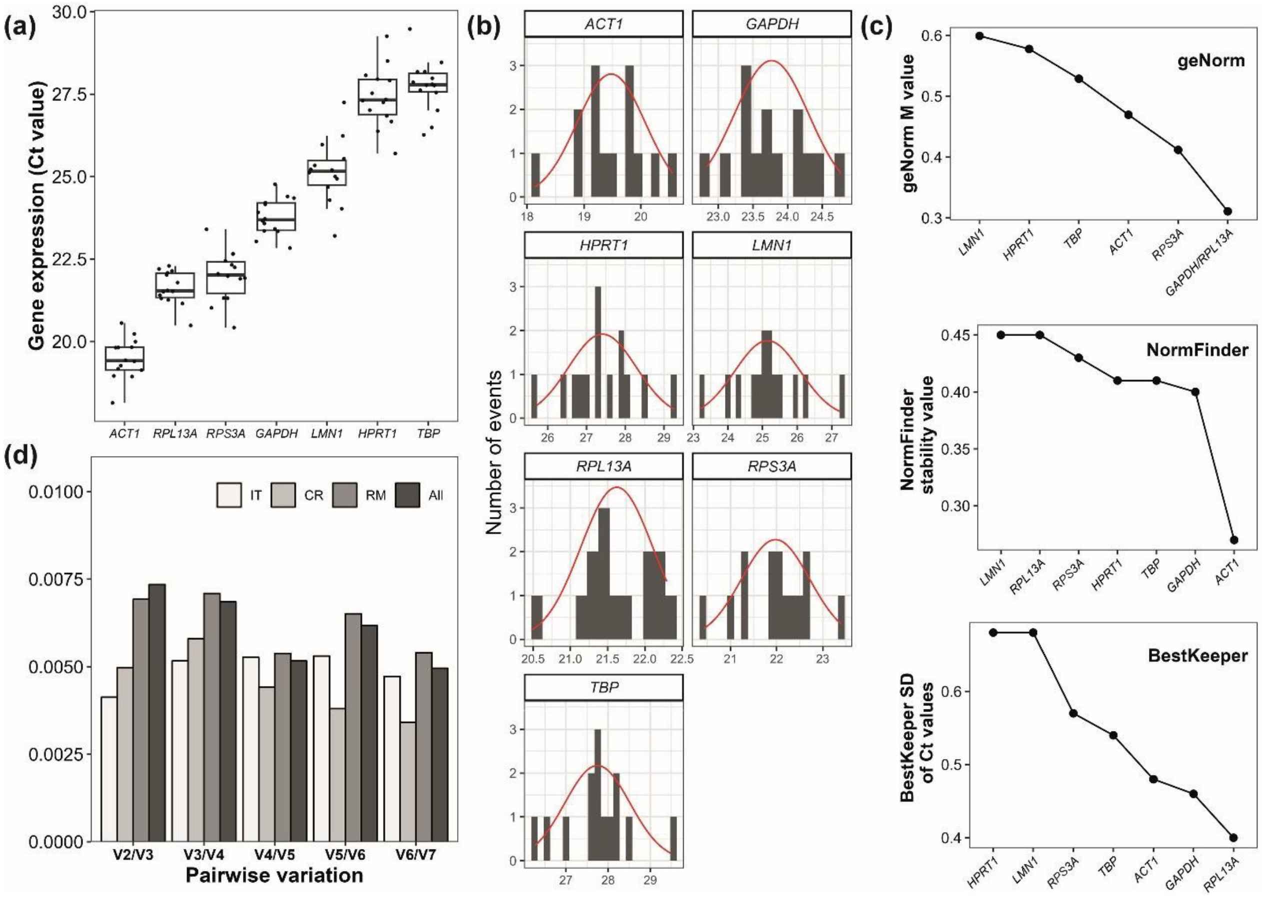

Figure 4. Expression variability and stability assessment of candidate reference genes in *Leucosolenia corallorrhiza*. (A) Boxplots showing the distribution of Ct values for the seven candidate reference genes across all samples, with lower Ct values indicating higher transcript abundance (Upper whisker extends from the top of the box (third quartile, Q3) to the most extreme data point within 1.5 times the Interquartile Range (IQR) above Q3. Lower whisker extends from the bottom of the box (first quartile, Q1) to the most extreme data point within 1.5 times the IQR below Q1). (B) Histograms with fitted curves illustrating the frequency distribution of the Ct values for each gene. (C) Stability ranking of the reference genes calculated using four algorithms: geNorm (M values), NormFinder (stability values), and BestKeeper (standard deviation of Ctvalues). Lower values correspond to higher stabilities. (D) Pairwise variation (V values) between combinations of reference genes across experimental groups (IT, intact tissue; CR, crescent/growing regenerative membrane; RM, fully sealed regenerative membrane; All, total dataset), as calculated using geNorm.

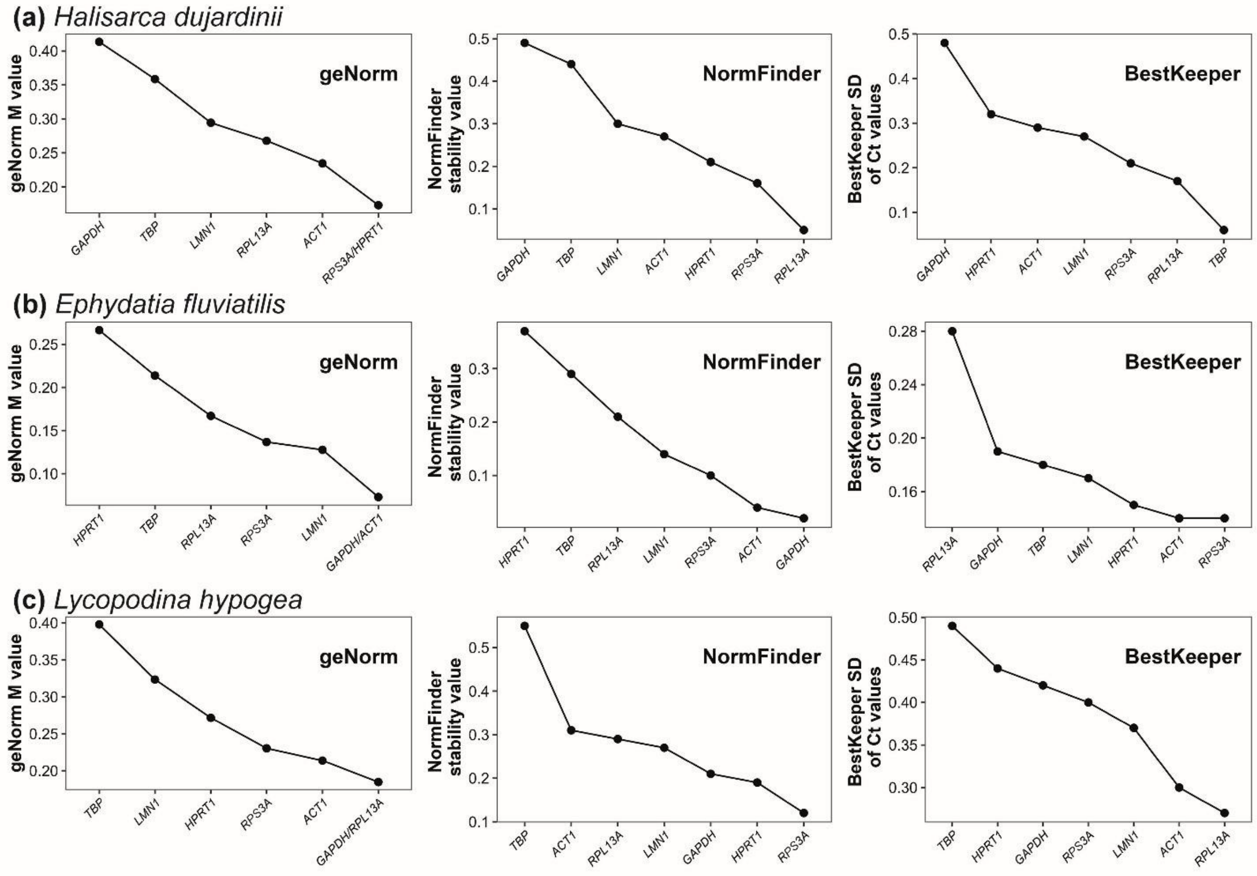

Figure 5. Stability ranking of candidate reference genes in the three sponge species based on three statistical algorithms. The expression stability of the seven candidate reference genes was evaluated in (A) *Halisarca dujardinii*, (B) *Ephydatia fluviatilis*, and (C) *Lycopodina hypogea* using geNorm (first column), NormFinder (second column), and BestKeeper (third column). Each graph shows the stability values or ranking scores for the tested genes, with lower values indicating a higher expression stability.

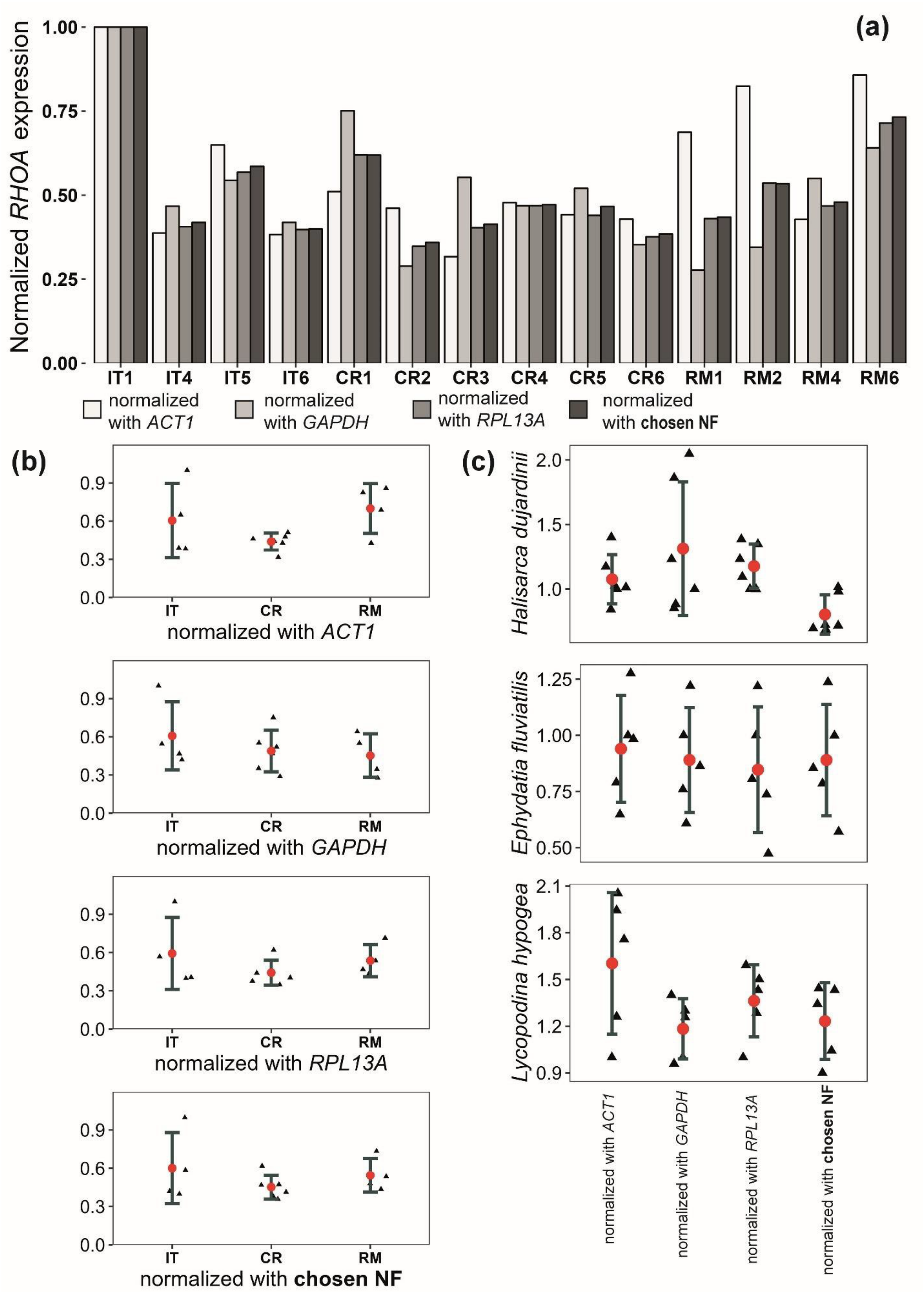

Figure 6. Impact of normalization strategy on *RHOA* expression estimated across sponge species. (A) Normalized *RHOA* gene expression in 3 experimental groups of

regeneration model (*L. corallorrhiza*); (B) scatter plots showing normalized *RHOA* mRNA expression for individual experiments in regeneration model of *L. corallorrhiza*; red dots represent mean value, whiskers represent standard deviation (SD) (C) Normalized *RHOA* mRNA expression for 3 other sponge species. Chosen NF is normalization factor, calculated as geometric mean of relative quantities of *GAPDH*, *ACTB* and *RPL13A*

Tables

Table 1. Cycle threshold (Ct) values and descriptive statistics for 7 candidate endogenous referent genes assayed across 14 samples from *Leucosolenia corallorrhiza* tissues.

Gene (*Lco-*)	Mean Ct	SD	CV	Min Ct	Median Ct	Max Ct	Range Ct	*Shapiro-Wilk. stat.*	*p-value*
ACT1	19.48	0.62	0.031	18.14	19.42	20.56	2.42	0.974	0.923
RPL13A	21.62	0.50	0.023	20.49	21.54	22.29	1.81	0.937	0.385
GAPDH	23.77	0.57	0.023	22.83	23.68	24.77	1.93	0.978	0.959
HPRT1	27.41	0.91	0.033	25.7	27.32	29.26	3.56	0.991	0.999
RPS3A	21.98	0.76	0.035	20.42	22.02	23.40	2.98	0.975	0.931
TBP	27.74	0.80	0.029	26.27	27.79	29.48	3.21	0.938	0.389
LMN1	25.14	0.98	0.039	23.20	25.16	27.25	4.05	0.974	0.921

Table 2. Stability ranking of candidate reference genes in sponge species according to GeNorm, NormFinder, and BestKeeper analysis.

Species	Ranking	1	2	3	Ranked top-3 at least in 2 of 3 algorithms
Leucosolenia corallorrhiza	GeNorm	GAPDH/RPL13A	RPS3A	ACT1	RPL13A, ACT1, GAPDH
	NormFinder	ACT1	GAPDH	TBP	
	BestKeeper	RPL13A	GAPDH	ACT1	
Halisarca dujardinii	GeNorm	RPS3A/HPRT1	ACT1	RPL13A	RPS3A, RPL13A, HPRT1
	NormFinder	RPL13A	RPS3A	HPRT1	
	BestKeeper	TBP	RPL13A	RPS3A	

Ephydatia fluviatilis	GeNorm	GAPDH/ACT1	LMN1	RPS3A	GAPDH, ACT1, RPS3A
	NormFinder	GAPDH	ACT1	RPS3A	
	BestKeeper	RPS3A	ACT1	HPRT1	
Lycopodina hypogea	GeNorm	GAPDH/RPL13A	ACT1	RPS3A	GAPDH, RPL13A, RPS3A, ACT1
	NormFinder	RPS3A	HPRT1	GAPDH	
	BestKeeper	RPL13A	ACT1	LMN1	

Supplementary material

Construction of the reference databases

The *de novo* transcriptome of *E. fluviatilis* was previously published (44). The *de novo* transcriptome of *L. corallorrhiza* was assembled using Trinity v2.15.2 with standard settings for paired-end reads and read normalization (45). The resulting transcriptome was processed using CD-HIT-EST (23), with a threshold of 0.95. The final assembly had 405255 transcripts with median and average contig lengths of 308 and 552, respectively; N50 metrics was 637. BUSCO score using eukaryota_odb10 BUSCO set was 98.4% (46).

Genome-based transcriptomes of *H. dujardini*i and *L. hypogea* were retrieved using publicly available genome assemblies (GCA_049997455.1 and GCA_963969325.1, respectively supplemented with our own RNA-seq data for gene annotation. Gene annotation was performed as reported previously (Borisenko et al., in press) using a combination of repeat masking (EarlGrey; (47)), ab initio predictors (BRAKER; (48-50), Trinity *de novo* and genome-guided transcriptome assemblies, StringTie (51) genome assemblies, and miniprot (52) alignment. The resulting genome-derived transcriptomes had BUSCO scores (eukaryota_odb10 BUSCO set) of 92.8 and 90.6%, respectively.

The reads used for the assemblies are available upon request. RNA was extracted using ExtractRNA (BC032, Evrogen, Moscow, Russia), followed by purification using a CleanRNA Standard kit (BC033, Evrogen, Moscow, Russia). Purified RNA samples in RNAse-free water were frozen and stored at -80°C until library preparation. The polyadenylated fraction of the total RNA was purified using the NEBNext Poly(A) mRNA Magnetic Isolation Module (New England Biolabs, Ipswich, MA, USA). Libraries were constructed using the NEBNext Ultra II RNA Library Prep Kit and sequenced using the Illumina NovaSeq6000 (Illumina, San Diego, CA, USA) in paired-end mode with 150 bp read length. The average read number per sample was 40 million. Reads were trimmed using fastp (53) and quality-checked using FastQC (Andrews S. (2010). FastQC: a quality control tool for high throughput sequence data. Available online at: http://www.bioinformatics.babraham.ac.uk/projects/fastqc).

The reads were obtained from three independent experimental sets. For *H. dujardinii,* 18 samples from a whole-body regeneration experiment were used (Melnikov et al., 2025) For *L. corallorrhiza*, we used 21 samples from unpublished body-wall regeneration experiments. For *L. hypoge*a, we used an unpublished dataset consisting of 3 samples of

intact tissues and 3 cultures of asexually produced buds. The details on the stages and number of replicates are reported in the Table S1.

Table S1. Sampling summary used in the RNA-seq experiments.

Species	Stage/physiological condition	No of replicates
Halisarca dujardinii	Intact tissues	3
	Primary multicellular aggregates	3
	Early-staged primmorphs	3
	True primmorphs	3
	Primmorphs with developing aquiferous system, early stage	3
	Fully reconstructed sponges	3
Leucosolenia corallorrhiza	Intact tissues	3
	30 min post-operation (po)	3
	1 h po	3
	3 h po	3
	6 h po	3
	12 h po	3
	24h po	3
	48h po	3
Lycopodina hypogea	Intact tissues	3
	Asexual buds	3

Table S2. Total list of transcripts used in the study.

Target	Species	Sequence name	NCBI/UniProt accesion	Top pblast hit (UniProtKB + SWISSProt)
GADPH	*Hdu*	Hdu_evm.model.contig25.17	/	A0A238BQ86_9BILA
	Efl	Efl_m.14207_g.14207	/	A0AA35RHM9_GEOBA
	Lhy	Lhy_evm.model.OZ017791.1.63	/	A0AA35RG30_GEOBA
	Lco	Lco_TRINITY_DN8809	/	A0A6I9YFP0_9SAUR
TBP	*Hdu*	Hdu_evm.model.contig90.47	/	A0A1X7VES3_AMPQE
	Efl	Efl_m.27899_g.27899	/	A0A1X7VES3_AMPQE
	Lhy	Lhy_evm.model.OZ017782.1.157	/	A0A1X7VES3_AMPQE

	Lco	TRINITY_DN57007_41916	/	A0A9J6E132_RHIMP
RPL13A	*Hdu*	Hdu_evm.model.contig30.236	/	A0AA35RN17_GEOBA
	Efl	Efl_m.11949_g.11949	/	A0AA35RN17_GEOBA
	Lhy	Lhy_evm.model.OZ017787.1.746	/	A0AA35RN17_GEOBA
	Lco	Lco_TRINITY_DN12876_134664	/	A0AA35RN17_GEOBA
RPS3A	*Hdu*	Hdu_HADA01000668.1	/	A0A6P8H362_ACTTE
	Efl	Efl_m.1283_g.1283	/	RS3A_SUBDO
	Lhy	Lhy_evm.model.OZ017787.1.307	/	RS3A_SUBDO
	Lco	Lco_TRINITY_DN9655_ 124502	/	A0A340YEV6_LIPVE
HPRT1	*Hdu*	Hdu_evm.model.contig22.67	/	A7STM7_NEMVE
	Efl	Efl_m.9314_g.9314	/	A0A1X7VL26_AMPQE
	Lhy	Lhy_evm.model.OZ017788.1.39	/	A0A3M6TC76_POCDA
	Lco	Lco_TRINITY_DN1202_3188	/	A0A3M6TC76_POCDA
RHOA	*Hdu*	Hdu_evm.model.contig87.143	/	A0A672JJP5_SALFA
	Efl	Efl_m.1105_g.1105	/	A0A1X7V1M3_AMPQE
	Lhy	Lhy_evm.model.OZ017790.1.75	/	A0AA35R3X6_GEOBA
	Lco	Lco_TRINITY_DN41272	/	A0AA88L6H2_ARTSF
LMN1	*Hdu*	Hdu_evm.model.contig32.21	/	A0AAN0INZ4_AMPQE
	Efl	Efl_m.31108_g.31108	/	A0AAN0INZ4_AMPQE
	Lhy	Lhy_evm.model.OZ017779.1.866	/	A0AAN0INZ4_AMPQE
	Lco	Lco_TRINITY_DN9368_99323	/	A0AAD7ZQB5_DIPPU
ACT1	*Hdu*	Hdu_evm.model.contig28.48	/	ACTC_PISOC
	Efl	Efl_m.16996_g.16996	/	A2SY09_CIOIN
	Lhy	Lhy_evm.model.OZ017783.1.600	/	A0A913Z1C1_PATMI
	Lco	Lco_TRINITY_DN24_95078	/	A7SCN8_NEMVE

Table S3. Description of all primers used to amplify candidate reference genes in the studied species. Primers labelled “F” are forward primers, and primers labelled “R” are reverse primers. Amplification efficiency was determined from each reference gene

primer set following RT-qPCR with five 1:10 serial dilutions. One result from three independent runs on different cDNA samples was used to evaluate PCR efficiency.

Gene	Primer sequence (5'-3')	PCR efficiency	Slope	Correlation coefficient (R^2)	T_a, °C
Lco-ACT1	F: GTACCACCATGTACCCTGGC<br>R: CGGACTCGTCATACTCCTGC	100,42%	-3,312	0,9993	57.0
Lco-RPL13A	F: CATTTGCTTGGTCGTCTGGC<br>R: TGATCTCCTCACACCGGACA	106.07%	-3,185	0,9979	59.9
Lco-GAPDH	F: AGGCGTACGGCATTACTGAG<br>R: AGGTCCACAACGGACACATC	99,99%	-3,322	0,9995	57.0
Lco-HPRT1	F: AGAACGTCGAAGAGCAGTGG<br>R: GTCGTCTTGCCGAACTCACT	100,16%	-3,318	0,9967	56.7
Lco-RPS3A	F: TCCGTCGCAAGATGGTTGAA<br>R: GATGGACTGGCAAGCCTTCT	102,61%	-3,261	0,9993	59.6
Lco-TBP	F: GTCAAGTTCTGGCACATCGC<br>R: AGGAGTTGCTGGCATCGAAA	99,75%	-3,328	0,9812	58.6
Lco-LMN1	F: TGCCGACGGACTGTTTATCC<br>R: CAGACCACCTTCCAGTGACC	100,97%	-3,300	0,9973	58.5
Lco-RHOA	F: CCAGTCAAGCCAGAGGAAGG<br>R: AGTTGCGACTTCGAACACCT	127,05%	-2,808	0,9959	57.1
Hdu-ACT1	F: GAGGCTCTCTTCCAGCCATC<br>R: TTCTGCATACGGTCGGCAAT	97.24%	-3.39	0,9997	58.4
Hdu-RPL13A	F: TGTCGTTCGGGGGATGATTC<br>R: AGGTGGCATGCCTTCAAAGA	104.66%	-3.215	0.9994	60.3
Hdu-GAPDH	F: GACCTCACCTGTCGCTTCAA<br>R: AGTGTAGCCCATCACTCCCT	101.35%	-3.29	0.9995	56.2
Hdu-HPRT1	F: GTTCTTCACCGACCTGCTCA<br>R: GCCCTCGTAGCTCTTCAGAC	99.45%	-3.335	0.9985	56.3
Hdu-RPS3A	F: AGTCACCAAAACCACTGGCA<br>R: CCCTACCCTGAACCTCCTCA	100.71%	-3.305	0.9998	57.9
Hdu-TBP	F: GGTTGGTAGCACCGATGTGA<br>R: CTCAGGCTCGTAACTGGCAA	97.83%	-3.375	0.9849	58.0
Hdu-LMN1	F: ACCCTTCGTGATGCAGACAG<br>R: GCGGTCCTTCAGTTCTTGGA	97.04%	-3.395	0.9977	58.2
Hdu-RHOA	F: CCTCCGAAACGACGAGAACA<br>R: CAAAGACTTCACGAACGCCC	99.87%	-3.325	0.9971	59.7
	F: CCGACAGGATGCAGAAGGAG	101.78%	-3.28	0.9990	58.0

Efl-ACT1	R: CACTGTACTTGCGCTCAGGA				
Efl-RPL13A	F: CTACCGCTTTGCGTGTTCTG<br>R: TTCTTGGAGCTGAGGCTTGG	103.09%	-3.25	0.9985	58.8
Efl-GAPDH	F: GACTTGACTGTGCGTCTGGA<br>R: CCTTGAGCTGGTCCGATTGT	99.04%	-3.345	0.9995	58.0
Efl-HPRT1	F: ATGGCTTCAGACAATGGGCA<br>R: CACACAGGGCTACAAGAGGG	98.23%	-3.365	0.9983	58.6
Efl-RPS3A	F: CAGCAATGTTTGCCGTCAGG<br>R: CGCGAGTGACACTTCGAAAA	99.87%	-3.325	0.9995	60.0
Efl-TBP	F: AGCACTCCCAGTTCTCAAGC<br>R: TACGTACCGGTCAGCACAAC	100.71%	-3.305	0.9990	55.8
Efl-LMN1	F: AGCTGGAAGATCAGGTTGCC<br>R: CCTTAACACACGACGCTCCT	99.45%	-3.335	0.9975	57.3
Efl-RHOA	F: GATGGTGCCTGTGGCAAAAC<br>R: AACACGGTGGGAACGTAGAC	102.43%	-3.265	0.9993	58.6
Lhy-ACT1	F: GGTCTGGTATGTGCAAGGCT<br>R: CGACCCACGATTGAAGGGAA	104.66%	-3.215	0.9995	58.4
Lhy-RPL13A	F: TGGTCCCACACAAGACCAAG<br>R: GCTTCAACTTCAGCACACGG	99.04%	-3.345	0.9994	58.4
Lhy-GAPDH	F: TCTTCACGACCACCGACAAG<br>R: ATGACCTTTGCCAGAGGAGC	100.71%	-3.305	0.9991	58.4
Lhy-HPRT1	F: TACTACTCCACAGCCAGCCA<br>R: ACCACCAGCACTGTCTTTCC	96.84%	-3.4	0.9928	56.2
Lhy-RPS3A	F: ACGAAAGGGAAGAAAGGGGC<br>R: GGGTTCTGGTCACAAGGGTT	99.04%	-3.345	0.9983	58.5
Lhy-TBP	F: ATCATTCGCAAGGTCTGCCA<br>R: GAAGCTGAAGCTGAGGGAGG	112.75%	-3.05	0.9978	58.8
Lhy-LMN1	F: CACCTACGATGCCAAGCTGA<br>R: TTCACAATCTGGTCGGTCGG	104.21%	-3.225	0.9976	59.7
Lhy-RHOA	F: CCGAGCAATGGCAGAGAAGA<br>R: TTGTTTGGAGAGCAGCACGA	99.25%	-3.34	0.9992	59.6

Table S4. Ranking of candidate reference genes in order of expression stability calculated by NormFinder. The numbers in brackets indicate stability values.

Ranking order	IT	CR	RM (*Lco-*)	All	IT (*Hdu-*)	IT (*Efl-*)	IT(*Lhy-*)

	(***Lco***-)	(***Lco***-)		(***Lco***-)			
1	*TBP* (0.23)	*RPS3A* (0.16)	*ACT1* (0.31)	*ACT1* (0.27)	*RPL13A* (0.05)	*GAPDH* (0.02)	*RPS3A* (0.12)
2	*RPS3A* (0.29)	*GAPDH* (0.18)	*HPRT1* (0.45)	*GAPDH* (0.40)	*RPS3A* (0.16)	*ACT1* (0.04)	*HPRT1* (0.19)
3	*HPRT1* (0.33)	*ACT1* (0.28)	*RPS3A* (0.49)	*HPRT1* (0.41)	*HRPT1* (0.21)	*RPS3A* (0.10)	*GAPDH* (0.21)
4	*RPL13A* (0.34)	*LMN1* (0.40)	*TBP* (0.51)	*TBP* (0.41)	*ACT1* (0.27)	*LMN1* (0.14)	*LMN1* (0.27)
5	*ACT1* (0.35)	*TBP* (0.41)	*RPL13A* (0.52)	*RPS3A* (0.43)	*LMN1* (0.30)	*RPL13A* (0.21)	*RPL13A* (0.29)
6	*GAPDH* (0.35)	*HPRT1* (0.64)	*LMN1* (0.55)	*LMN1* (0.45)	*TBP* (0.44)	*TBP* (0.29)	*ACT1* (0.31)
7	*LMN1* (0.40)	*RPL13A* (0.74)	*GAPDH* (0.59)	*RPL13A* (0.45)	*GAPDH* (0.49)	*HPRT1* (0.37)	*TBP* (0.55)

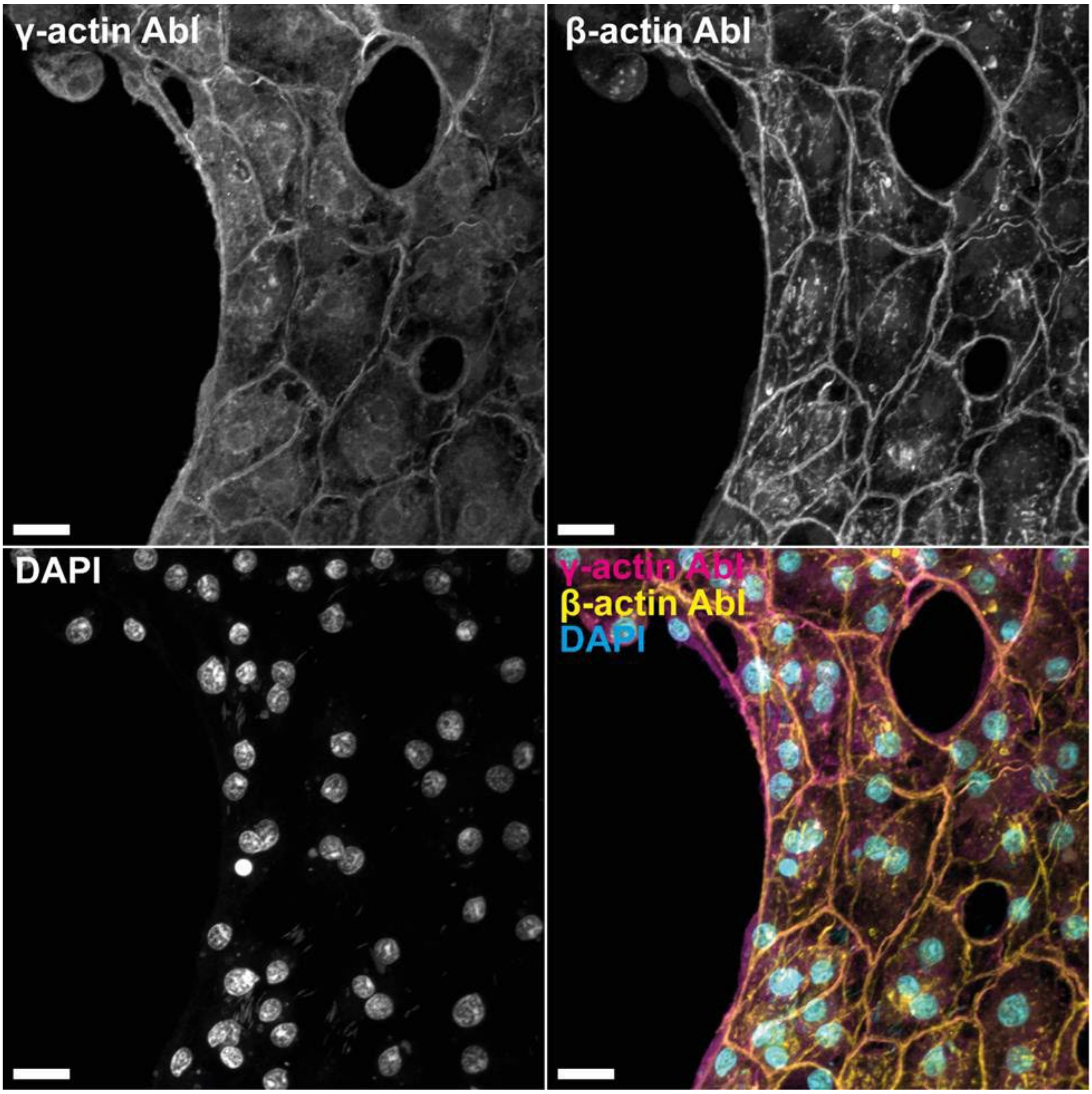

Figure S1. Growing regenerative membrane, *Leucosolenia corallorrhiza*, ~18-22 hours post operation, exo- and endopinacocytes are displayed. MIP of CLSM image. Scale bar 10μm. Cyan – DAPI (D9564, Sigma Aldrich), magenta – mouse anti-actin-γ antibodies (BioRad MCA 5776GA, clone 2A3), yellow – polyclonal rabbit anti-actin-β antibodies (ab8227, Abcam).

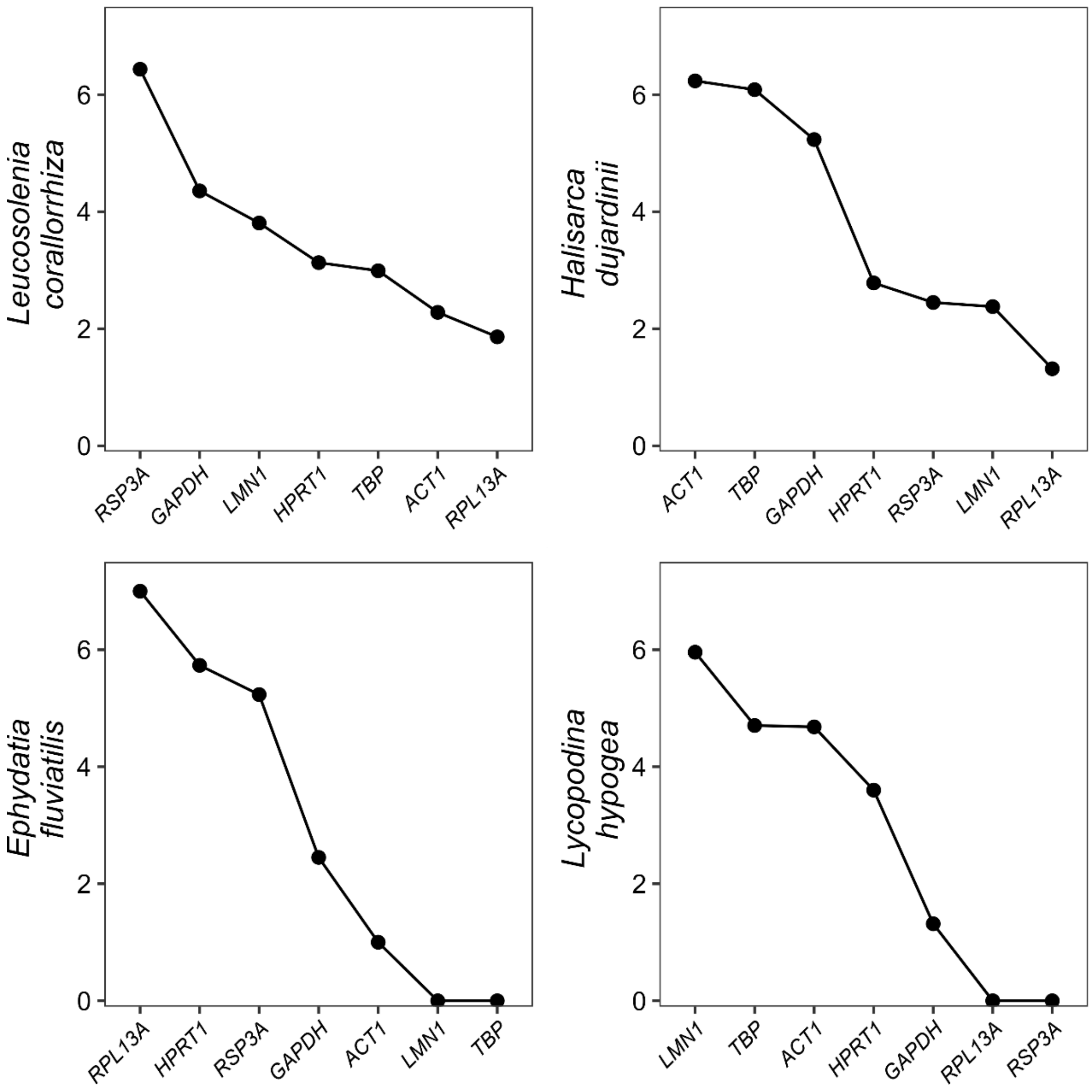

Figure S2. RefFinder stability rankings for candidate reference genes in four sponge species. Rankings are shown for *Leucosolenia corallorrhiza*, *Halisarca dujardinii*, *Ephydatia fluviatilis*, and *Lycopodina hypogea*. The y-axis represents the RefFinder stability score (lower values indicate higher stability according to the algorithm). Note the biologically implausible ranking of TBP as the most stable gene in *E. fluviatilis*, despite its relatively high variability in other stability analyses.

References

1. Bustin SA, Benes V, Garson JA, Hellemans J, Huggett J, Kubista M, et al. The MIQE guidelines: minimum information for publication of quantitative real-time PCR experiments. Clinical chemistry. 2009;55(4):611-22.
2. Hruz T, Wyss M, Docquier M, Pfaffl MW, Masanetz S, Borghi L, et al. RefGenes: identification of reliable and condition specific reference genes for RT-qPCR data normalization. BMC Genomics. 2011;12(1):156.
3. Schmittgen TD, Zakrajsek BA. Effect of experimental treatment on housekeeping gene expression: validation by real-time, quantitative RT-PCR. Journal of Biochemical and Biophysical Methods. 2000;46(1):69-81.
4. Pfaffl MW. A new mathematical model for relative quantification in real-time RT–PCR. Nucleic Acids Research. 2001;29(9):e45-e.
5. Vandesompele J, De Preter K, Pattyn F, Poppe B, Van Roy N, De Paepe A, et al. Accurate normalization of real-time quantitative RT-PCR data by geometric averaging of multiple internal control genes. Genome Biology. 2002;3(7):research0034.1.
6. Chapman JR, Waldenström J. With Reference to Reference Genes: A Systematic Review of Endogenous Controls in Gene Expression Studies. PloS one. 2015;10(11):e0141853.
7. Selvey S, Thompson EW, Matthaei K, Lea RA, Irving MG, Griffiths LR. β-Actin—an unsuitable internal control for RT-PCR. Molecular and Cellular Probes. 2001;15(5):307-11.
8. Ereskovsky A, Lavrov A. Porifera. Invertebrate Histology2021. p. 19-54.
9. Adamska M. Differentiation and Transdifferentiation of Sponge Cells. In: Kloc M, Kubiak JZ, editors. Marine Organisms as Model Systems in Biology and Medicine. Cham: Springer International Publishing; 2018. p. 229-53.
10. Leys SP, Riesgo A. Epithelia, an evolutionary novelty of metazoans. Journal of experimental zoology Part B, Molecular and developmental evolution. 2012;318(6):438-47.
11. Riesgo ANA, PÉRez-Porro AR, Carmona S, Leys SP, Giribet G. Optimization of preservation and storage time of sponge tissues to obtain quality mRNA for next-generation sequencing. Molecular Ecology Resources. 2012;12(2):312-22.
12. Melnikov NP, Bolshakov FV, Frolova VS, Skorentseva KV, Ereskovsky AV, Saidova AA, et al. Tissue homeostasis in sponges: Quantitative analysis of cell proliferation and apoptosis. Journal of experimental zoology Part B, Molecular and developmental evolution. 2022;338(6):360-81.
13. Musser JM, Schippers KJ, Nickel M, Mizzon G, Kohn AB, Pape C, et al. Profiling cellular diversity in sponges informs animal cell type and nervous system evolution. Science (New York, NY). 2021;374(6568):717-23.
14. Riesgo A, Farrar N, Windsor PJ, Giribet G, Leys SP. The analysis of eight transcriptomes from all poriferan classes reveals surprising genetic complexity in sponges. Molecular biology and evolution. 2014;31(5):1102-20.
15. Ruperti F, Dziecìatkowska M, Pankey MS, Asensio CS, Anselmetti D, Fernàndez-Busquets X, et al. Proteomic analysis of the sponge Aggregation Factor implicates an ancient toolkit for allorecognition and adhesion in animals. Proceedings of the National Academy of Sciences of the United States of America. 2024;121(52):e2409125121.
16. Affenzeller S, Cerveau N, Jackson DJ. Identification and validation of reference genes for qPCR in the terrestrial gastropod Cepaea nemoralis. PloS one. 2018;13(8):e0201396.
17. Li X, Gong P, Wang B, Wang C, Li M, Zhang Y, et al. Selection and validation of experimental condition-specific reference genes for qRT-PCR in Metopolophium dirhodum (Walker) (Hemiptera: Aphididae). Scientific reports. 2020;10(1):21951.
18. Seneca FO, Forêt S, Ball EE, Smith-Keune C, Miller DJ, van Oppen MJ. Patterns of gene expression in a scleractinian coral undergoing natural bleaching. Marine biotechnology (New York, NY). 2010;12(5):594-604.

19. Basu S, Pereira AE, Pinheiro DH, Wang H, Valencia-Jiménez A, Siegfried BD, et al. Evaluation of reference genes for real-time quantitative PCR analysis in southern corn rootworm, Diabrotica undecimpunctata howardi (Barber). Scientific reports. 2019;9(1):10703.
20. Liu Z, Xiao J, Xia Y, Wu Q, Zhao C, Li D. Selection and validation of reference genes for RT-qPCR-based analyses of Anastatus japonicus Ashmead (Hymenoptera: Helicopteridae). Frontiers in Physiology. 2022;Volume 13 - 2022.
21. Pantile R, Webster N. Strict thermal threshold identified by quantitative PCR in the sponge Rhopaloeides odorabile. Marine Ecology Progress Series. 2011;431:97-105.
22. Lavrov A, Ekimova I, Schepetov D, Koinova A, Ereskovsky A. The complex case of the calcareous sponge Leucosolenia complicata (Porifera: Calcarea): hidden diversity in Boreal and Arctic regions with description of a new species. Zoological Journal of the Linnean Society. 2023;200(4):876-914.
23. Camacho C, Coulouris G, Avagyan V, Ma N, Papadopoulos J, Bealer K, et al. BLAST+: architecture and applications. BMC Bioinformatics. 2009;10(1):421.
24. Duvaud S, Gabella C, Lisacek F, Stockinger H, Ioannidis V, Durinx C. Expasy, the Swiss Bioinformatics Resource Portal, as designed by its users. Nucleic Acids Res. 2021;49(W1):W216-w27.
25. Edgar RC. MUSCLE: a multiple sequence alignment method with reduced time and space complexity. BMC Bioinformatics. 2004;5:113.
26. Capella-Gutiérrez S, Silla-Martínez JM, Gabaldón T. trimAl: a tool for automated alignment trimming in large-scale phylogenetic analyses. Bioinformatics (Oxford, England). 2009;25(15):1972-3.
27. Trifinopoulos J, Nguyen LT, von Haeseler A, Minh BQ. W-IQ-TREE: a fast online phylogenetic tool for maximum likelihood analysis. Nucleic Acids Res. 2016;44(W1):W232-5.
28. Skorentseva KV, Bolshakov FV, Saidova AA, Lavrov AI. Regeneration in calcareous sponge relies on 'purse-string' mechanism and the rearrangements of actin cytoskeleton. Cell and tissue research. 2023;394(1):107-29.
29. Skorentseva KV, Bolshakov FV, Saidova AA, Lavrov AI. Microtubule organization and tubulin post-translational modifications in intact tissues and during regeneration in calcareous sponges. Cell and tissue research. 2025;400(3):365-81.
30. Xie F, Wang J, Zhang B. RefFinder: a web-based tool for comprehensively analyzing and identifying reference genes. Functional & integrative genomics. 2023;23(2):125.
31. Kozera B, Rapacz M. Reference genes in real-time PCR. Journal of applied genetics. 2013;54(4):391-406.
32. Casas AI, Hassan AA, Manz Q, Wiwie C, Kleikers P, Egea J, et al. Un-biased housekeeping gene panel selection for high-validity gene expression analysis. Scientific reports. 2022;12(1):12324.
33. Djari A, Madignier G, Chervin C, van der Rest B, Giovannoni JJ, Bouzayen M, et al. A stable combination of non-stable genes outperforms standard reference genes for RT-qPCR data normalization. Scientific reports. 2024;14(1):31278.
34. de Jonge HJ, Fehrmann RS, de Bont ES, Hofstra RM, Gerbens F, Kamps WA, et al. Evidence based selection of housekeeping genes. PloS one. 2007;2(9):e898.
35. Young AP, Landry CF, Jackson DJ, Wyeth RC. Tissue-specific evaluation of suitable reference genes for RT-qPCR in the pond snail, Lymnaea stagnalis. PeerJ. 2019;7:e7888.
36. Volland M, Blasco J, Hampel M. Validation of reference genes for RT-qPCR in marine bivalve ecotoxicology: Systematic review and case study using copper treated primary Ruditapes philippinarum hemocytes. Aquatic toxicology (Amsterdam, Netherlands). 2017;185:86-94.
37. Ding D, Sun XJ, Yan M, Chen Q, Gao L, Kang CJ. The ECSIT Mediated Toll3-Dorsal-ALFs Pathway Inhibits Bacterial Amplification in Kuruma Shrimp. Frontiers in immunology. 2022;13:807326.
38. Lockyer AE, Spinks J, Kane RA, Hoffmann KF, Fitzpatrick JM, Rollinson D, et al. Biomphalaria glabrata transcriptome: cDNA microarray profiling identifies resistant- and

susceptible-specific gene expression in haemocytes from snail strains exposed to Schistosoma mansoni. BMC Genomics. 2008;9:634.
39. Barber RD, Harmer DW, Coleman RA, Clark BJ. GAPDH as a housekeeping gene: analysis of GAPDH mRNA expression in a panel of 72 human tissues. Physiological Genomics. 2005;21(3):389-95.
40. Ruan W, Lai M. Actin, a reliable marker of internal control? Clinica Chimica Acta. 2007;385(1):1-5.
41. Sun Y, Li Y, Luo D, Liao DJ. Pseudogenes as weaknesses of ACTB (Actb) and GAPDH (Gapdh) used as reference genes in reverse transcription and polymerase chain reactions. PloS one. 2012;7(8):e41659.
42. De Spiegelaere W, Dern-Wieloch J, Weigel R, Schumacher V, Schorle H, Nettersheim D, et al. Reference gene validation for RT-qPCR, a note on different available software packages. PloS one. 2015;10(3):e0122515.
43. de Seze J, Gatin J, Coppey M. RhoA regulation in space and time. FEBS letters. 2023;597(6):836-49.
44. Alié A, Hayashi T, Sugimura I, Manuel M, Sugano W, Mano A, et al. The ancestral gene repertoire of animal stem cells. Proceedings of the National Academy of Sciences of the United States of America. 2015;112(51):E7093-100.
45. Grabherr MG, Haas BJ, Yassour M, Levin JZ, Thompson DA, Amit I, et al. Full-length transcriptome assembly from RNA-Seq data without a reference genome. Nature biotechnology. 2011;29(7):644-52.
46. Simão FA, Waterhouse RM, Ioannidis P, Kriventseva EV, Zdobnov EM. BUSCO: assessing genome assembly and annotation completeness with single-copy orthologs. Bioinformatics (Oxford, England). 2015;31(19):3210-2.
47. Baril T, Galbraith J, Hayward A. Earl Grey: A Fully Automated User-Friendly Transposable Element Annotation and Analysis Pipeline. Molecular biology and evolution. 2024;41(4).
48. Gabriel L, Brůna T, Hoff KJ, Ebel M, Lomsadze A, Borodovsky M, et al. BRAKER3: Fully automated genome annotation using RNA-seq and protein evidence with GeneMark-ETP, AUGUSTUS, and TSEBRA. Genome research. 2024;34(5):769-77.
49. Brůna T, Lomsadze A, Borodovsky M. GeneMark-ETP significantly improves the accuracy of automatic annotation of large eukaryotic genomes. Genome research. 2024;34(5):757-68.
50. Huang N, Li H. compleasm: a faster and more accurate reimplementation of BUSCO. Bioinformatics (Oxford, England). 2023;39(10).
51. Kovaka S, Zimin AV, Pertea GM, Razaghi R, Salzberg SL, Pertea M. Transcriptome assembly from long-read RNA-seq alignments with StringTie2. Genome Biology. 2019;20(1):278.
52. Li H. Protein-to-genome alignment with miniprot. Bioinformatics (Oxford, England). 2023;39(1).
53. Chen S. Ultrafast one-pass FASTQ data preprocessing, quality control, and deduplication using fastp. iMeta. 2023;2(2):e107.

Click here to access/download
Supporting Information
Skorentseva2026_Supplementary material.docx
