# Supplementary material for: Validating the classics: Accurate reference gene panel for reliable RT-qPCR in Porifera
Source: PLoS One. 2026 May 20;21(5):e0349507. doi: 10.1371/journal.pone.0349507 (PMC13189343; doi:10.1371/journal.pone.0349507)
Supplement: Attachment — Submitted filename: comments.docx [file pone.0349507.s003.docx]

Line 22-33 Based on the experiment design and goals in M&M, the authors tested all candidate housekeeping genes from L. corallorrhiza on all four distinctive species to see if these housekeeping genes from L. corallorrhiza are universal for other three species. However, the abstract does not clearly verify this main goals, please reorganize Abstract.

Line 104-105 Please add this ‘hpo’ into the list of abbreviations used in manuscript

Line 105 and Line 116 Authors need to elaborate why sample size for IT (n=4), CR (24 hpo, n=6) and RM (48 hpo, n=4) is different.

Line 105-

Line 134 please give how much tissue in gram was used for RNA extraction per preparation per replicate

Line 134-137 and Line 139-142 repeated information, please adjust this. It is more suitable for subsection of ‘RNA extraction and reverse transcription”

Line 144-145 please add citation of RNA extraction method.

Line 144-162 please reorganize this section

Line 165-166 ‘b)’ were used for different transcriptome databases

Line 167-168 assemblies in Table S1 is not publicly available, it is upon the request, not sure if this is against PlosOne’s policy

Line 168 Please list the name and accession number of the query amino acid sequence used in search. What are criteria used in search ?

Line 174 ‘For lamins” ? please give more information of this in introduction section

Line 174-182 Please clarify this section. What is purpose of having this section here ? I believe you retrieved and used sequences from other species to search homologies targeted for housekeeping genes and validation gene from transcriptome databases of various sponge species, then rewrite this section.

Line 182 authors said the ‘accession IDs’ are available in Table S2 for NCBI and Uniprot, however the access ID for NCBI is empty.

Line 183-184 what is the size of amplicon for each target gene with designed primers ?

Line 199 Ta ? you mean Tm ?

Line 201-202 this sentence needs to be clarified

Line 204 if you use standard software with qPCR equipment, the software calculates the primer efficiency for you.

Line 232 genus name of each species could be abbreviated as *H*. , *E*. and *L*. as they were mentioned in previous sections

Line 242-243 Figure 1 does not match the results as said. Same to Figure 2 and Figure 3, double check the Figure legend.

Line 284 in Figure 4a panel, the letter assigned to each subpanel should be consistent with one in Figure legend, either in lower case or upper case. This should apply to other Figures too.

Line 298-327 all knowledge information of four analysis methods can be combined and move to introduction.

Line 310 For Figure 4d, please add values and conduct statistical comparisons. Same for Figure 6a.

Line 316-20 this part is more suitable for introduction.

Line 383-404 anova (statistical analysis) for multacomparisons are needed here to address and support your results.

Line 406 in discussion section, authors emphasized on the validation of housekeeping genes and their stability across species and tissue, this is one the key point of this manuscript. Meanwhile, the sequence comparisons and phylogenetic analysis prior to the candidate housekeeping gene selection is another key point authors brought into this manuscript, this makes this research unique and more valuable to scientists. Please elaborate this part more.

Line 494 In limitations section, authors listed a few potential issues when apply this protocol into other studies, which should be mentioned in abstract in a few words, such as ‘suitability of housekeeping genes should be reevaluated when …. before …”

For Figures

Please increase the font size to make them readable. Also add data at each data point in Figures.
