# Supplementary material for: Validating the classics: Accurate reference gene panel for reliable RT-qPCR in Porifera
Source: PLoS One. 2026 May 20;21(5):e0349507. doi: 10.1371/journal.pone.0349507 (PMC13189343; doi:10.1371/journal.pone.0349507)
Supplement: Attachment — Submitted filename: Response to Reviewers_final.docx [file pone.0349507.s006.docx]

**Manuscript ID:** ONE-D-26-03346

Title: Validating the classics: accurate reference gene panel for reliable RT-qPCR in Porifera

PLOS One

Authors: Kseniia V. Skorentseva, Nikolai P. Melnikov, Alexander V. Ereskovsky, Andrey I. Lavrov & Alina A. Saidova

*Please submit your revised manuscript by Apr 08 2026 11:59PM. If you will need more time than this to complete your revisions, please reply to this message or contact the journal office at* [*plosone@plos.org*](mailto:plosone@plos.org)*. When you're ready to submit your revision, log on to https://www.editorialmanager.com/pone/ and select the 'Submissions Needing Revision' folder to locate your manuscript file.*

*Please include the following items when submitting your revised manuscript:*

- *A letter that responds to each point raised by the academic editor and reviewer(s). You should upload this letter as a separate file labeled 'Response to Reviewers'.*
- *A marked-up copy of your manuscript that highlights changes made to the original version. You should upload this as a separate file labeled 'Revised Manuscript with Track Changes'.*
- *An unmarked version of your revised paper without tracked changes. You should upload this as a separate file labeled 'Manuscript'.*

**Journal Requirements:**

When submitting your revision, we need you to address these additional requirements.

1. Please ensure that your manuscript meets PLOS ONE's style requirements, including those for file naming. The PLOS ONE style templates can be found at

https://journals.plos.org/plosone/s/file?id=wjVg/PLOSOne_formatting_sample_main_body.pdf and <https://journals.plos.org/plosone/s/file?id=ba62/PLOSOne_formatting_sample_title_authors_affiliations.pdf>

The manuscript has been reformatted in accordance with the journal’s formatting guidelines and templates provided.

2.  Thank you for stating the following financial disclosure: “The research was supported by the Russian Science Foundation project no. 23–74–10005 (studies in *Leucosolenia corallorrhiza* and *Halisarca dujardinii*) and no. 24-14-00452 (studies in *Ephydatia fluviatilis* and *Lycopodina hypogea*).”

Please state what role the funders took in the study. If the funders had no role, please state: "The funders had no role in study design, data collection and analysis, decision to publish, or preparation of the manuscript."

If this statement is not correct you must amend it as needed. Please include this amended Role of Funder statement in your cover letter; we will change the online submission form on your behalf.

We have added the mentioned statement to the Funding section of the manuscript.

3. In the online submission form, you indicated that “A draft dataset supporting the conclusions of this study is available upon request. The R script used to analyze and visualize the data is also available.”

All PLOS journals now require all data underlying the findings described in their manuscript to be freely available to other researchers, either 1. In a public repository, 2. Within the manuscript itself, or 3. Uploaded as supplementary information. This policy applies to all data except where public deposition would breach compliance with the protocol approved by your research ethics board. If your data cannot be made publicly available for ethical or legal reasons (e.g., public availability would compromise patient privacy), please explain your reasons on resubmission and your exemption request will be escalated for approval.

In accordance with PLOS ONE’s data availability policy, we have deposited the raw dataset underlying the findings of this study, as well as the R script used for data analysis and visualization, in a public repository (Mendeley Data).

The materials are available at the following DOI: 10.17632/stkgvs9s26.1

The Data Availability Statement in the manuscript has been updated accordingly.

4. When completing the data availability statement of the submission form, you indicated that you will make your data available on acceptance. We strongly recommend all authors decide on a data sharing plan before acceptance, as the process can be lengthy and hold up publication timelines. Please note that, though access restrictions are acceptable now, your entire data will need to be made freely accessible if your manuscript is accepted for publication. This policy applies to all data except where public deposition would breach compliance with the protocol approved by your research ethics board. If you are unable to adhere to our open data policy, please kindly revise your statement to explain your reasoning and we will seek the editor's input on an exemption. Please be assured that, once you have provided your new statement, the assessment of your exemption will not hold up the peer review process.

We confirm that the data underlying the findings of this study have now been made fully and freely available in a public repository (Mendeley Data).

The Data Availability Statement in both the manuscript and the online submission form has been updated accordingly to reflect that the data are publicly accessible at the provided DOI.

5. Please upload a new copy of Figure 2 as the detail is not clear. Please follow the link for more information:  <https://journals.plos.org/plosone/s/figures>

We have uploaded a new version of Figure 2 in higher resolution to ensure that all details are clearly visible.

6. We notice that your supplementary [figures/tables] are included in the manuscript file. Please remove them and upload them with the file type 'Supporting Information'. Please ensure that each Supporting Information file has a legend listed in the manuscript after the references list.

We have removed all supplementary figures and tables from the main manuscript file and uploaded them separately under the file type “Supporting Information,” as requested. The files and manuscript have been updated accordingly.

7. If the reviewer comments include a recommendation to cite specific previously published works, please review and evaluate these publications to determine whether they are relevant and should be cited. There is no requirement to cite these works unless the editor has indicated otherwise.

We confirm that the reviewer comments did not contain any specific recommendations to cite previously published works. The suitable references have been added to the revised manuscript where appropriate.

**Reviewers' comments:**

**Reviewer's Responses to Questions**

**Comments to the Author**

1. Is the manuscript technically sound, and do the data support the conclusions?

The manuscript must describe a technically sound piece of scientific research with data that supports the conclusions. Experiments must have been conducted rigorously, with appropriate controls, replication, and sample sizes. The conclusions must be drawn appropriately based on the data presented.

Reviewer #1: Yes

Reviewer #2: Yes

Reviewer #3: Yes

2. Has the statistical analysis been performed appropriately and rigorously?

Reviewer #1: Yes

Reviewer #2: No

Reviewer #3: Yes

3. Have the authors made all data underlying the findings in their manuscript fully available?

The [PLOS Data policy](http://www.plosone.org/static/policies.action#sharing) requires authors to make all data underlying the findings described in their manuscript fully available without restriction, with rare exception (please refer to the Data Availability Statement in the manuscript PDF file). The data should be provided as part of the manuscript or its supporting information, or deposited to a public repository. For example, in addition to summary statistics, the data points behind means, medians and variance measures should be available. If there are restrictions on publicly sharing data—e.g. participant privacy or use of data from a third party—those must be specified.

Reviewer #1: Yes

Reviewer #2: Yes

Reviewer #3: Yes

4. Is the manuscript presented in an intelligible fashion and written in standard English?

PLOS ONE does not copyedit accepted manuscripts, so the language in submitted articles must be clear, correct, and unambiguous. Any typographical or grammatical errors should be corrected at revision, so please note any specific errors here.

Reviewer #1: Yes

Reviewer #2: Yes

Reviewer #3: Yes

5. Review Comments to the Author

Please use the space provided to explain your answers to the questions above. You may also include additional comments for the author, including concerns about dual publication, research ethics, or publication ethics. (Please upload your review as an attachment if it exceeds 20,000 characters).

We thank Reviewers for their positive comments. We appreciate the time they took to critically review the manuscript and to make recommendations that have significantly improved our manuscript. We have accepted majority of recommendations and hope that these revisions address the Reviewers’ criticisms.

**Reviewer #1:** This study evaluated the stability of seven reference gene candidates selected for RT-qPCR normalization in gene expression quantification, specifically for the four different sponge species. The work provided a panel of reference genes for RT-qPCR in species belonging to phylum of Porifera. It can be accepted for publication after the following questions are addressed and included in discussion as appropriate:

We sincerely thank the reviewer for their positive assessment and constructive comments. We have carefully considered all the reviewer’s suggestions and have addressed them in the revised manuscript. Relevant points have been incorporated into the Discussion section as appropriate.

1. Only one species of sponges from each type (marine, freshwater, filter-feeding and carnivorous form) was used for validation, do you expect that other species of the same type will result in similar reference gene stability profile?

We thank the reviewer for this insightful question.

As noted, the sponge species used in this study cannot be strictly classified into four distinct types. Instead, the four categories considered (marine, freshwater, filter-feeding, and carnivorous) represent combinations of these characteristics between different species: among the species used, there is one representative of calcareous sponges and three representatives of demosponges; additionally, there are one freshwater species, three marine species, three filter-feeding species, and a carnivorous one. Thus, each species combines these traits in different ways.

We agree that using a single representative species for each ecological or functional group (marine, freshwater, filter-feeding, and carnivorous) does not allow us to fully generalize the stability profiles to all species within these categories. However, our study was specifically designed to provide an initial cross-species validation across phylogenetically and ecologically diverse representatives of Porifera, rather than to establish universally stable reference genes for entire groups. Importantly, our results already indicate that no single gene is universally stable even across the four tested species, despite their grouping by ecological type. Instead, we observed that while the exact rankings vary, a consistent subset of genes (e.g., *RPL13A*, *ACT1*, *GAPDH*, and *RPS3A*) repeatedly appears among the most stable candidates across species. This suggests that, although species-specific validation remains necessary, there is a conserved core of relatively stable reference genes that can serve as a starting point for further studies. Based on these observations, we do not expect identical stability rankings in other species of the same ecological type. Rather, we expect that: (i) stability profiles will remain species-specific, and (ii) the same set of candidate genes is likely to perform robustly after validation, even if their ranking order differs. To emphasize this point, we have revised the Discussion section of the manuscript accordingly.

2. Many studies are conducted to examine gene expressions under specific stress conditions. How well do the results presented in this study translate to gene expression studies under conditions such as thermal stress, starvation, exposure to heavy metals, etc.?

We agree that reference gene stability can be influenced by experimental conditions such as thermal stress, starvation, or exposure to pollutants, and therefore results obtained under baseline conditions cannot be directly extrapolated to all stress contexts. In the present study, our goal was to identify candidate reference genes that are stable under standard physiological conditions across phylogenetically and ecologically diverse sponge species, thereby providing a baseline framework for normalization. Our results show that even under non-stressed conditions, there is no universally stable gene across species, highlighting the necessity of careful validation. Based on this, we expect that under stress conditions, gene expression variability may further increase, potentially altering the stability ranking of candidate reference genes. Therefore, we do not assume that the exact stability profiles identified here will remain unchanged under conditions such as thermal stress, starvation, or exposure to heavy metals. At the same time, we observed that several genes (e.g., *RPL13A*, *ACT1*, *GAPDH*, and *RPS3A*) consistently rank among the most stable candidates across multiple species. This suggests that these genes may represent a robust starting panel for reference gene selection, including in stress-related experiments. However, we emphasize that their suitability must be empirically validated under each specific experimental condition. We have now clarified this point in the Discussion, explicitly stating that environmental and physiological stressors may affect reference gene stability and that additional validation is required when applying these genes to stress-response studies and also added a corresponding statement to the Conclusion section of the manuscript to reflect this point.

3. How representative are the specimens from each species in representing the entire species?

All specimens used in the study were typical representatives of the corresponding species in sense of overall body structure, physiological condition (only specimens considered fully healthy was used in the study), habitat, etc. Culturing conditions for *L. hypogea* and gemmules of *E. fluvatilis* are well accepted and routinely used. The specimens of each species were indeed collected from relatively limited geographical areas, and we have added a description of this limitation to the ‘Limitations’ section of the manuscript. Nevertheless, we believe that this does not compromise the overall validity of our findings, as such a limitation is common in organismal biology studies conducted on non-model organisms.

4. For Figure 4c, 5, and S2, why are the data presented in dot plots with connected lines instead of bar graphs for presentation when reference gene stability ranking is not continuous? Please double check to see if the current data presentation is appropriate.

We have chosen to present the data in dot plots with connected lines following the approach used in a large body of previously published studies of this type [17, 39, 40, 46. We believe that this style of visualization facilitates comparison of data across different studies for future researchers.

**Reviewer #2:**

We sincerely thank Reviewer 2 for their thorough and careful evaluation of our manuscript. We greatly appreciate the detailed and constructive comments, which have helped us improve the clarity and quality of our work.

1. Line 22-33 Based on the experiment design and goals in M&M, the authors tested all candidate housekeeping genes from *L. corallorrhiza* on all four distinctive species to see if these housekeeping genes from *L. corallorrhiza* are universal for other three species. However, the abstract does not clearly verify these main goals, please reorganize Abstract.

We have revised the Abstract to clearly reflect the main goals of the study, including the testing of candidate housekeeping genes from *L. corallorrhiza* across the other three species, in accordance with the experimental design described in the Materials and Methods.

2. Line 104-105 Please add this ‘hpo’ into the list of abbreviations used in manuscript

We defined the used term upon first mention. The term is also listed in the abbreviations.

3. Line 105 and Line 116 Authors need to elaborate why sample size for IT (n=4), CR (24 hpo, n=6) and RM (48 hpo, n=4) is different.

The differences in sample size (IT, n = 4; CR at 24 hpo, n = 6; RM at 48 hpo, n = 4) were due to technical limitations encountered during sample processing. cDNA synthesis was performed only from samples with sufficiently high RNA concentration and quality, which resulted in unequal final sample numbers across experimental groups.

We have clarified this point in the revised manuscript.

4. Line 105-Line 134 please give how much tissue in gram was used for RNA extraction per preparation per replicate

The exact mass of tissue used for RNA extraction per replicate was not measured. Instead, normalization of samples within each species was performed at the cDNA synthesis stage by using equal amounts of RNA for reverse transcription. This has been clarified in lines 181-182.

5. Line 134-137 and Line 139-142 repeated information, please adjust this. It is more suitable for subsection of ‘RNA extraction and reverse transcription”

The repeated information has been removed and the text has been reorganized accordingly. The relevant methodological details have been incorporated into the subsection “RNA extraction and reverse transcription,” as suggested.

6. Line 144-145 please add citation of RNA extraction method.

The following citation has been added to the manuscript Chomczynski P, Sacchi N. Single-step method of RNA isolation by acid guanidinium thiocyanate–phenol–chloroform extraction. Anal Biochem. 1987;162(1):156–9. doi:10.1006/abio.1987.9999. [26]

7. Line 144-162 please reorganize this section

The section corresponding to Lines 144–162 has been reorganized, taking into account all the revisions mentioned above.

8. Line 165-166 ‘b)’ were used for different transcriptome databases

This typographical error has been corrected in the revised manuscript.

9. Line 167-168 assemblies in Table S1 is not publicly available, it is upon the request, not sure if this is against PlosOne’s policy

The assemblies used for searching target genes/transcripts are essentially draft assemblies and are yet to be published in dedicated studies. We believe that the fasta files with target nucleotide sequences and primer sequences (Table S3) are sufficient for reproducing the results of the current study – regarding both the results of RT-PCR and the validation of targets’ identities.

Line 168 Please list the name and accession number of the query amino acid sequence used in search. What are criteria used in search?

We added queries’ names and IDs to the table S2 Regarding the criteria, we used the default tblastn parameters implemented in BioEdit with BLOSUM62 matrix.

10. Line 174 ‘For lamins”? please give more information of this in introduction section

A brief description of lamins, including their role in nuclear structure and chromatin organization, has been added to the M&M section, in the context of the approach used in this study and also included the information about genes selected for this study in introduction.

11. Line 174-182 Please clarify this section. What is purpose of having this section here? I believe you retrieved and used sequences from other species to search homologies targeted for housekeeping genes and validation gene from transcriptome databases of various sponge species, then rewrite this section.

This section refers specifically to ML analysis of the phylogeny of intermediate filaments. We wanted to verify that the sequences found via blast are indeed nuclear lamins and not a result of contamination (e.g., nematode cytoplasmic IFs that often show the presence of lamin domains). We believe that the details provided in the section are necessary to reproduce the analysis.

Line 182 authors said the ‘accession IDs’ are available in Table S2 for NCBI and Uniprot, however the access ID for NCBI is empty.

The missing NCBI accession IDs were due to the fact that the corresponding nucleotide sequences had not yet been deposited in a public repository at the time of submission. To ensure transparency and data accessibility, we have now provided a separate FASTA file containing all referenced nucleotide sequences in the Supplementary Information. The manuscript and Table S2 have been updated accordingly.

12. Line 183-184 what is the size of amplicon for each target gene with designed primers?

The amplicon sizes for each target gene with the designed primers have been added to Table S3 in the revised manuscript.

Line 199 Ta? you mean Tm?

In the Materials and Methods, we report the practical annealing temperature (Ta) used for the reaction, rather than the theoretically calculated melting temperature (Tm). This reflects the actual conditions applied during the PCR experiments.

13. Line 201-202 this sentence needs to be clarified

The sentence has been clarified to ensure that the description in the Materials and Methods section is consistent with the embedded figures.

14. Line 204 if you use standard software with qPCR equipment, the software calculates the primer efficiency for you.

Due to technical considerations and our careful approach to data analysis, we chose to use external software to calculate primer efficiencies rather than relying on the standard qPCR equipment software.

15. Line 232 genus name of each species could be abbreviated as H., E. and L. as they were mentioned in previous sections

The suggested corrections have been implemented in the revised manuscript.

16. Line 242-243 Figure 1 does not match the results as said. Same to Figure 2 and Figure 3, double check the Figure legend.

All figures and their legends have been carefully checked and corrected to ensure they accurately correspond to the reported images.

17. Line 284 in Figure 4a panel, the letter assigned to each subpanel should be consistent with one in Figure legend, either in lower case or upper case. This should apply to other Figures too.

The lettering of all figure panels has been standardized to ensure consistency with the figure legends throughout the manuscript.

18. Line 298-327 all knowledge information of four analysis methods can be combined and move to introduction.

We appreciate the reviewer’s suggestion. To improve the clarity and logical flow of the manuscript, we have added a concise description of the four analytical methods to the Introduction, where the overall analytical framework is first introduced. However, we prefer to retain this section in its current position, as presenting the description of the four analysis methods alongside the corresponding results facilitates clarity and improves the overall readability for the readers. We thank the reviewer for this helpful suggestion.

19. Line 310 For Figure 4d, please add values and conduct statistical comparisons. Same for Figure 6a.

Unfortunately, statistical analysis of the data presented in Figures 4d and 6a is not applicable, as the values shown do not represent biological replicates or independent sample sets suitable for statistical comparison, but results of calculations. To improve transparency, we have provided the numerical values used to generate these graphs in Table S4 and Table S6 in the Supplementary Information.

20. Line 316-20 this part is more suitable for introduction.

See the answer to comment 18

21. Line 383-404 anova (statistical analysis) for multacomparisons are needed here to address and support your results.

In this section, our aim was not to demonstrate statistically significant differences in expression levels resulting from normalization using different reference genes, but rather to illustrate the reduction in variance achieved through multi-gene normalization. Specifically, our intention was to show that normalization using multiple validated reference genes decreases the standard deviation, thereby improving the reliability of downstream statistical analyses and facilitating the detection of truly significant expression differences. To ensure transparency, we have now provided the numerical data used to make Figures 6b and 6c in Table 3.

22. Line 406 in discussion section, authors emphasized on the validation of housekeeping genes and their stability across species and tissue, this is one the key point of this manuscript. Meanwhile, the sequence comparisons and phylogenetic analysis prior to the candidate housekeeping gene selection is another key point authors brought into this manuscript, this makes this research unique and more valuable to scientists. Please elaborate this part more.

We thank the reviewer for this important comment and for highlighting this aspect of our study. We have expanded the Discussion to emphasize this point and to clarify how this approach contributes to the robustness and broader applicability of our results

23. Line 494 In limitations section, authors listed a few potential issues when apply this protocol into other studies, which should be mentioned in abstract in a few words, such as ‘suitability of housekeeping genes should be reevaluated when …. before …”

A brief statement reflecting the limitations of applying this protocol to other studies has been added to the Abstract, highlighting that the suitability of housekeeping genes should be reevaluated when used under different conditions or in other species.

***For Figures***

Please increase the font size to make them readable. Also add data at each data point in Figures.

The figures have been reorganized, and the font sizes have been increased to improve readability. The numerical values have been added to the data points in Fig 4c and 5 as requested.

**Reviewer #3:**

Skorentseva et al used transcriptomic data sets to identify a suite of candidate reference genes based on similarity with known sequences and evaluated their suitability for poriferan RT-qPCR analyses. The manuscript is well-written, the methods employed are appropriate, and both the results and conclusions are supported by the data shown. While the study will undoubtedly prove to be a useful framework for future gene expression studies, the authors might consider more fully contextualizing how they envision their findings can be applied. In addition, it would be helpful if the authors expanded on their methodological details, particularly the immunostaining shown in Figure S1, which does not appear to be described in the Methods. More detailed comments are listed below.

We are grateful to the reviewer for their insightful and supportive comments. Their thoughtful feedback has helped us improve the manuscript and clarify both the applicability of our findings and the methodological details.

***Specific comments***

Methodological details.

1. - Provide accession numbers/gene names for the sequences used to query the reference databases.
- Define the criteria used to trim sequence reads with TrimAI.

We provided sequence names and UniProt IDs in the Table S2. Regarding the TrimAl, we changed the line to include “--automated1” parameter used for trimming. This parameter is recommended by TrimAl developers as most suited for ML analysis [30].

2. - Were the qPCR products sequence validated?

The qPCR products were not sequenced. However, specificity was confirmed by analyzing melting curves to ensure that each primer pair amplified a single product. In addition, particular attention was paid during primer design to minimize the likelihood of off-target amplification.

3. - Provide methodological details for the immunostaining shown in Figure S1. Relatedly, the text on pg 9 references two antibodies for actin B, but only the polyclonal antibody is shown in Figure S1.

The detailed immunostaining protocol has been previously published [32, 33]. We have updated Figure S1 to include images obtained with the monoclonal antibodies for β-actin (BioRad MCA5775GA, clone 4C2), and the corresponding text in the manuscript has been revised accordingly to reflect these changes (Lines 299-302).

4. Pg 9, 2nd paragraph – Expression analysis of the candidate reference genes was evaluated across “all samples”. It would be helpful if the term “all samples” was more explicitly defined.

A clarification has been added to the manuscript to explicitly define the abovementioned phrase.

5. pg 10 – “TBP and LMN1 exhibited the highest variability”. However, Figure 4C shows that the lowest values were observed for LMN1 and HPRT1.

The typo has been corrected to accurately represent the variability of TBP, LMN1, and HPRT1 as shown in Fig 4c.

6. Clarify what sample sets/cDNAs were used to evaluate the candidate reference genes in the other species. Were the same time points/tissues as those referenced in Figure 4 used?

As described in the Materials and Methods section (lines 117-138), for the other species, cDNA was prepared from total RNA extracted from lysates of appropriate number of intact individuals of the corresponding species.

7. The results section describing data shown in Figure 6a indicates that the pattern of the NF primers relative to the individual primers was consistent across all experimental conditions. The IT1 sample set, however, does not show this pattern. Is this because the results were normalized to this dataset? If so, please state that in the Figure legend.

The results for Figure 6a were indeed normalized to the IT1 sample set. This clarification has been added to the figure legend to accurately reflect the normalization procedure.

6) Unless it is a journal stipulation, the authors might consider moving the limitations section further up or incorporating the limitations directly into the Discussion.

In response, we have moved the limitations section into the Discussion and integrated it with the main interpretation of the results.

7) Specifically label the y-axis in Figure 4B, 6B, and 6C.

The y-axis labels of Figures 4B, 6B, and 6C have been updated accordingly to clearly indicate the measured values.

***Minor comments***

1) pg 2, 2nd to last line – check the spelling of Porifera

The typo has been corrected.

2) pg 3 - delete the spurious quote at the of the Introduction

The typo has been corrected.

3) pg 4 – define “hpo”; place a space between the integer value and the unit abbreviation (eg 0.5 mM rather than 0.5mM)

The abbreviation “hpo” has been defined, and spaces have been added between numerical values and unit abbreviations throughout the manuscript.

4) pg 5 – change “were soaked at” to “were dabbed on…”; change “0.1-10 ug of total RNA…” to “Total RNA (01-1 ug) was taken…”

The suggested changes have been incorporated into the manuscript.

5) pg 6 – change “Retrieved transcripts were…” to “The retrieved Porifera transcripts were…”

The suggested changes have been incorporated into the manuscript.

6) pg 7 – it is unclear if Ta is meant to be the annealing temp of the various primers, please define. Also, indicate that the primer specific temperatures are listed in Table S3.

The requested changes clarifying this issue have been incorporated.

7) pg 7 – change to “In this model, …”

The suggested changes have been incorporated into the manuscript.

8) pg 8 – change to “we aligned candidate transcripts to…cytoplasmic isoforms of vertebrate actin”

The suggested changes have been incorporated into the manuscript.

9) pg 9 – change to “To validate that the identified contigs encode cytoplasmic actins in sponges, *L. corallhorrihiza* regenerative membranes were immunostained with antibodies targeting various actins. Subsequent imaging revealed that cytoplasmic filamentous structures and stress fibers were stained with a certain level of differentiation between the different actin isoforms.” NOTE – the specific antibodies used can be moved to the methods section.

The requested changes to the text have been incorporated. The detailed immunostaining protocol has been previously published and is referenced in the manuscript.

10) pg 11 – change to “…and RPS3A were among the most stably expressed…”

The suggested changes have been incorporated into the manuscript.

11) Figure 3 legend – something seems be missing in the parentheses

That typo has been corrected.

6. PLOS authors have the option to publish the peer review history of their article ([what does this mean?](https://journals.plos.org/plosone/s/editorial-and-peer-review-process#loc-peer-review-history)). If published, this will include your full peer review and any attached files.

If you choose “no”, your identity will remain anonymous but your review may still be made public.

**Do you want your identity to be public for this peer review?** For information about this choice, including consent withdrawal, please see our [Privacy Policy](https://www.plos.org/privacy-policy).

Reviewer #1: No

Reviewer #2: No

Reviewer #3: No
